# Internet-Delivered Psychological Treatments for Mood and Anxiety Disorders: A Systematic Review of Their Efficacy, Safety, and Cost-Effectiveness

**DOI:** 10.1371/journal.pone.0098118

**Published:** 2014-05-20

**Authors:** Filip K. Arnberg, Steven J. Linton, Monica Hultcrantz, Emelie Heintz, Ulf Jonsson

**Affiliations:** 1 Department of Neuroscience, Psychiatry, Uppsala University, Uppsala, Sweden; 2 Center for Health and Medical Psychology, Department of Law, Psychology, and Social Work, Örebro University, Örebro, Sweden; 3 Swedish Council on Health Technology Assessment, Stockholm, Sweden; 4 Center for Medical Technology Assessment (CMT), Department of Medical and Health Sciences, Linköping University, Linköping, Sweden; 5 Division of Insurance Medicine, Department of Clinical Neuroscience, Karolinska Institutet, Stockholm, Sweden; University of Melbourne, Australia

## Abstract

**Background:**

Greater access to evidence-based psychological treatments is needed. This review aimed to evaluate whether internet-delivered psychological treatments for mood and anxiety disorders are efficacious, noninferior to established treatments, safe, and cost-effective for children, adolescents and adults.

**Methods:**

We searched the literature for studies published until March 2013. Randomized controlled trials (RCTs) were considered for the assessment of short-term efficacy and safety and were pooled in meta-analyses. Other designs were also considered for long-term effect and cost-effectiveness. Comparisons against established treatments were evaluated for noninferiority. Two reviewers independently assessed the relevant studies for risk of bias. The quality of the evidence was graded using an international grading system.

**Results:**

A total of 52 relevant RCTs were identified whereof 12 were excluded due to high risk of bias. Five cost-effectiveness studies were identified and three were excluded due to high risk of bias. The included trials mainly evaluated internet-delivered cognitive behavioral therapy (I-CBT) against a waiting list in adult volunteers and 88% were conducted in Sweden or Australia. One trial involved children. For adults, the quality of evidence was graded as moderate for the short-term efficacy of I-CBT vs. waiting list for mild/moderate depression (*d* = 0.83; 95% CI 0.59, 1.07) and social phobia (*d* = 0.85; 95% CI 0.66, 1.05), and moderate for no efficacy of internet-delivered attention bias modification vs. sham treatment for social phobia (*d* = −0.04; 95% CI −0.24, 0.35). The quality of evidence was graded as low/very low for other disorders, interventions, children/adolescents, noninferiority, adverse events, and cost-effectiveness.

**Conclusions:**

I-CBT is a viable treatment option for adults with depression and some anxiety disorders who request this treatment modality. Important questions remain before broad implementation can be supported. Future research would benefit from prioritizing adapting treatments to children/adolescents and using noninferiority designs with established forms of treatment.

## Introduction

A pressing challenge for mental health services is meeting the demand for the treatment of depression and anxiety disorders. Nearly 40% of the population is estimated to be in need of treatment at some time during their life for anxiety or depression [1]. Each year 14–18% of the population across the age span suffer an anxiety disorder and 7–9% suffer from depression in the United States as well as in Europe [1], [2]. Thus, meeting the needs of people suffering anxiety and depression with the current delivery methods is a gargantuan task [3]–[5].

Only one third of depressed patients respond fully to pharmacotherapy [6] and patients prefer psychological to pharmacologic treatment for depression and anxiety at a 3∶1 rate [7]. Fortunately, cognitive behavioral treatments are helpful for anxiety and depression for adults [8]–[10] and for children and adolescents [11]. Other psychological therapies such as interpersonal and psychodynamic therapies have also been reported to produce significant improvements [10], [12], [13]. However, limited access to qualified therapists restricts the utility of psychological treatments. In fact, of those with a serious problem as many as 50% in developed and 85% in undeveloped countries will simply go untreated [14]. Of those who do receive treatment, rates of quality care are moderate to low for anxiety disorders [15].

The internet has offered a new avenue for providing psychological treatments, but the effectiveness of these treatments is still an issue. Most reviews to date have found support for the use of internet-delivered cognitive-behavioral therapy (I-CBT) [16]–[18]. For example, a meta-analytical review found that I-CBT was helpful for four distinct disorders [19]. Similarly, Hedman et al. [20] reviewed randomized controlled trials (RCTs) of I-CBT and reported large effects for depression, social phobia and panic disorder. While ambitious, extant reviews nevertheless fail to address some key issues.

First, the quality of the evidence needs to be carefully considered. In previous reviews, when used at all, quality assessments were restricted to a few indices of the internal validity of the individual studies. A proper assessment of risk of bias is essential to avoid the risk of drawing false conclusions, however, and it can be justified to exclude studies of higher risk of bias from the synthesis [21]. A recent example is a Cochrane review that found moderate clinical effect of exercise on depression when including all relevant trials regardless of risk of bias [22]. However, when restricting the analysis to the trials with low risk of bias, the estimate indicated only a small effect of exercise that did not reach statistical significance.

Furthermore, investigators that conduct systematic reviews and meta-analyses are increasingly aware that not only individual studies but also the body of evidence needs to be systematically evaluated, because the confidence in the pooled effect estimates may be compromised not only by risk of bias in individual studies but also by several other factors (e.g., imprecision, inconsistency, indirectness, and publication bias) [23]. The issue of quality assessment is compounded further if reviews are conducted by the trial authors themselves [20], [24]. For example, the Cochrane Collaboration requires an independent assessment of eligibility and risk of bias by a second author not involved in the study/studies due to potential conflicts of interest [25]. Also, as experts in the content area under review they may have pre-formed opinions that can influence their assessments [26]. Given that the extant reviews were conducted by the trial authors themselves, the field would gain additional credibility from an independent evaluation.

Second, the issue of noninferiority has been largely ignored in previous reviews, but is necessary when comparing an existing evidence-based treatment (e.g., CBT) with a new one (e.g., I-CBT). In contrast to investigations of psychological therapy that involve new methods in areas where there is no known evidence-based treatment, the internet programs wisely employ known treatment techniques; only the manner of treatment delivery is altered. A greater reach and eventual cost savings could make internet therapies viable alternatives in healthcare. A critical issue, then, is whether they are noninferior to existing treatment. Noninferiority trials have gained increased attention to help in clinical decision making as the list of possible treatments grows, since a new treatment should be at least not inferior to existing evidence-based ones [27]. The methodology for noninferiority trials differ from superiority trials [27] and there is a need to review the literature from this perspective. Previous reviews on internet-delivered treatments generally conclude that these treatments have effects equivalent to the established forms of treatments [18]–[20], [28]. However, the absence of a significant difference between two treatments in a clinical trial is not the same as a proof of noninferiority. Furthermore, formal indirect comparisons of treatment effect estimates between trials are only appropriate if the new and established treatments were compared against a reference that is similar both in methods and population [29], which, in this case seems to be a indeterminate presumption. We therefore believe that the field is ripe for an analysis that elaborates on the issue of noninferiority vs. superiority.

Noninferiority trials are difficult to design and execute well [27]. Circumstances that strengthen inferences about superiority, because they increase similarities across treatment arms, can have the reverse effect on inferences of noninferiority. If a novel treatment is in fact inferior to established treatments, a trial with a sloppy design will be biased against finding this difference [27]. Superiority trials mainly use intention-to-treat (ITT) samples whereas noninferiority should be demonstrated also in the per-protocol analysis because an ITT analysis tends to dilute differences. Furthermore, there should be a fairness of comparisons between the new and established treatment, such that the established treatment is implemented rigorously under conditions that do not compromise the assay sensitivity. For example, if many subjects in a trial have previously failed to respond to the control treatment, there would be a bias in favor of the new treatment [30]. Noninferiority trials could also provide data for whether internet therapies are cost-effective, with important implications for healthcare.

Third, the previous reviews have largely ignored potential adverse events (e.g., harms, side effects, and deterioration), which may prove important for implementation of remotely delivered psychological treatments. Finally, reviews to date have focused on CBT, while trials of other treatments have begun to emerge [31].

The current review addresses all of the above issues. It has been conducted under the auspices of the Swedish Council on Health Technology Assessment (SBU), a government agency that has produced numerous systematic reviews evaluating the effects of various treatments (www.sbu.se/en/). The overall aim of this report is to provide a systematic review of the literature evaluating internet-delivered psychological treatment for mood and anxiety disorders with attention to methodological quality, consideration of the noninferiority perspective, and with ratings of the quality of the evidence using Grading of Recommendations Assessment, Development and Evaluation (GRADE) [23] by a freestanding council. Specifically, the following questions guided the review (additional questions were addressed in the governmental report):

1. Is internet-delivered psychological treatment efficacious, safe and cost-effective for mood and anxiety disorders in children, adolescents and adults?
2. Is internet-delivered treatment noninferior to established psychological treatments?

## Methods

### Protocol and registration

This systematic review was conducted at SBU. The inclusion criteria were pre-specified and a protocol was registered in advance internally at SBU (ref. no UTV2012/26), see Protocol S1.

### Eligibility criteria

Only published studies in English were considered for this review. The criteria for eligibility included the following characteristics.

#### Patients

Children, adolescents and adults with anxiety or mood disorders according to the manuals of the American Psychiatric Association [32] and the World Health Organization [33]. The specific diagnoses included were major depressive disorder, dysthymia, bipolar disorder, social phobia, panic disorder, generalized anxiety disorder (GAD), posttraumatic stress disorder (PTSD), obsessive-compulsive disorder (OCD), specific phobia, and separation anxiety (in children and adolescents). Studies were excluded if the participants were selected primarily because of a specific physical illness.

#### Interventions

Internet-delivered psychological treatments, defined as interventions based on an explicit psychological theory, not conducted at a clinic, and delivered to the patients via the internet. Any support had to be remotely delivered (e.g. email-like messages or telephone). The degree of support was categorized into pure self-help (no support), technician-assisted (e.g., non-clinical), or therapist-guided (i.e., clinical support).

#### Comparator

Any established psychological treatments, waiting list, usual care, or attention control.

#### Outcome

Change in symptoms of the primary disorder, adverse events, and cost per effect and per quality-adjusted life-years.

#### Study design

For short-term effects and risk of adverse events only RCTs were included. For long-term follow-up assessments (i.e., ≥6 months after post-assessment) RCTs and observational studies were included because of the ethical and practical dilemmas of conducting long-term RCTs. For cost-effectiveness data, economic evaluations based on individual-level data and decision models were eligible.

### Information sources

Electronic searches were conducted using Medical Subject Headings (MeSH) and relevant text word terms. The databases used were PubMed, Cochrane Library, CINAHL, PsycINFO, Psychology and Behavioral Sciences Collection (PBSC), TRIP database and CRD, up to March 4, 2013.

### Search strategy

We used search terms for depression/mood and anxiety and for each disorder (e.g., panic, phobia), for a range of delivery methods (e.g., online, internet, web, computer, phone), and for therapy, psychotherapy, intervention, and terms for specific interventions (e.g., cognitive behavioral, psychodynamic, interpersonal). The detailed search strategies are found in Appendix S1.

### Study selection

Two reviewers independently screened the titles and abstracts identified by the search strategy. All studies of potential relevance according to the inclusion criteria were obtained in full text and two reviewers independently assessed them for inclusion. Any disagreements were resolved by discussions. Reference lists were screened for additional studies of relevance. Appendix S2 lists the efficacy and cost-effectiveness reports that were excluded after full-text reading.

### Data collection process

From each included study of moderate or low risk of bias (see below), data was extracted and inserted in a table by one reviewer. A second reviewer audited the data extraction. Any disagreements were resolved by discussion.

### Data items

Information was extracted from included trials on (1) participants (age, education, diagnosis, and method of diagnostic assessment); (2) treatment (including treatment paradigm, level of support, duration); (3) type of comparator (4) outcome measures of core symptoms; (5) adverse events or deterioration; (6) costs.

### Risk of bias in individual studies

Two reviewers independently assessed the risk of bias with the use of checklists developed for each relevant study design [34]. Risk of bias is the systematic tendency that any aspect of the study makes the estimated treatment effect deviate from its true value, that is, the extent to which results of an included trial can be believed. The checklist for RCTs used hereinis highly similar to the Cochrane Collaboration's tool for assessing risk of bias [26] and includes 31 items to consider for the randomization (methods and outcome; 3 items), treatment (blinding, compliance, therapists, confounds; 5 items) and assessment (blinding, reliability, validity, timing, analysis; 9 items) of the participants, dropout (size, balance, covariates, analysis; 5 items), reporting bias (protocol, primary/secondary outcome, adverse events, assessment, 6 items), and conflicts of interest (3 items). A rating of low, moderate or high risk of bias was given to each category of items and was combined into a global rating of the trial.

Trials that had a serious flaw were rated as high risk of bias; trials that met all or nearly all criteria were rated as low risk of bias, such as trials with a convincing comparator (e.g., an established treatment or a sham versions of attention bias modification) and no other obvious risk of bias; the remainder were rated as moderate risk of bias. Trials of moderate risk vary in their strengths and weaknesses: some trials likely provide valid results while others are only possibly valid. A high-risk trial is not valid; the results are at least as likely to reflect flaws in the study design as true differences among the trial arms. A fatal flaw may be reflected by one aspect introducing a high risk of bias or by failure to meet combinations of item criteria. The reviewers agreed on rules-of-thumb for decisions on categories and alert attention to trials that had, for example, *N*<30, dropout >20%, or unbalanced baseline characteristics. We included trials for the evaluation of long-term effects if they had a dropout rate of less than 30% and reported on other treatments during the follow-up period. For health-economic studies to be included they had to report both costs and effects. Any disagreements were resolved by consensus or by arbitration by a third reviewer. If necessary, study authors were solicited to provide additional information. Only studies with low or moderate risk of bias were used for further synthesis.

### Planned method of analysis

We included as noninferiority designs all comparisons of internet-delivered treatment vs. established psychological therapies (e.g. I-CBT compared with individual therapist-led CBT). For these comparisons we used a predefined noninferiority margin of *d* = −0.2, chosen because it relates to a small effect size [35] and to ensure that noninferior treatments would retain an advantage over no treatment. All other comparisons were evaluated as superiority designs. Meta-analyses were carried out in RevMan 5. The calculations of the standardized mean differences were based on the groups' sample sizes, means and standard deviations at post-treatment. If the number of participants at post-treatment were not reported, the group sizes at randomization were used. Random effects models were used. All effect sizes in this report refer to between-group effects. Costs were converted to USD and the 2013 price-level [36].

### Publication bias

Potential publication bias was assessed for plausibly effective interventions by inspecting funnel plots and by a trim-and-fill procedure [37], which yields an estimate of the effect size after taking bias into account (analyses performed in Comprehensive Meta-Analysis v2, Biostat Inc.).

### Quality of evidence (GRADE)

The international grading system GRADE [23] was used to assess the quality of evidence for effects and safety with regard to groups of studies relevant to each treatment and support type, population, and disorder, according to the following four levels:

- High quality (⊕⊕⊕⊕) –We are very confident that the true effect lies close to that of the estimate of the effect.
- Moderate quality (⊕⊕⊕○) –We are moderately confident in the effect estimate: The true effect is likely to be close to the estimate of the effect, but there is a possibility that it is substantially different.
- Low quality (⊕⊕○○)–Our confidence in the effect estimate is limited: The true effect may be substantially different from the estimate of the effect.
- Very low quality (⊕○○○) – We have very little confidence in the effect estimate: The true effect is likely to be substantially different from the estimate of the effect.

In the GRADE system, evidence based on RCTs begins as high quality evidence, but may be rated down for several reasons, including study limitations, inconsistency of results, indirectness of evidence, imprecision or reporting bias. That is, for each type of treatment and support type, for each disorder and population, the quality of the evidence was assumed to be high at the outset, but subsequently rated down if there were limitations in the relevant studies. For example, there were three trials of CBT with clinical support vs. waiting list for adult participants diagnosed with panic disorder. Evidence of treatment efficacy start as being of high quality because the trials were RCTs, while study limitations (waiting list comparison [WLC]), inconsistency in the results (two trials show favorable effect, one shows no effect), and imprecision across studies (all three trials have small samples) entailed that the body of evidence finally received a low-quality rating. The quality of evidence was decided upon through discussions among the authors and input from an external group, the Quality and Priority Group at the agency. In line with agency guidelines we rated down for indirectness when only one RCT was included for a specific question, unless the included RCT was a multi-center trial.

## Results

**Figure 1:**
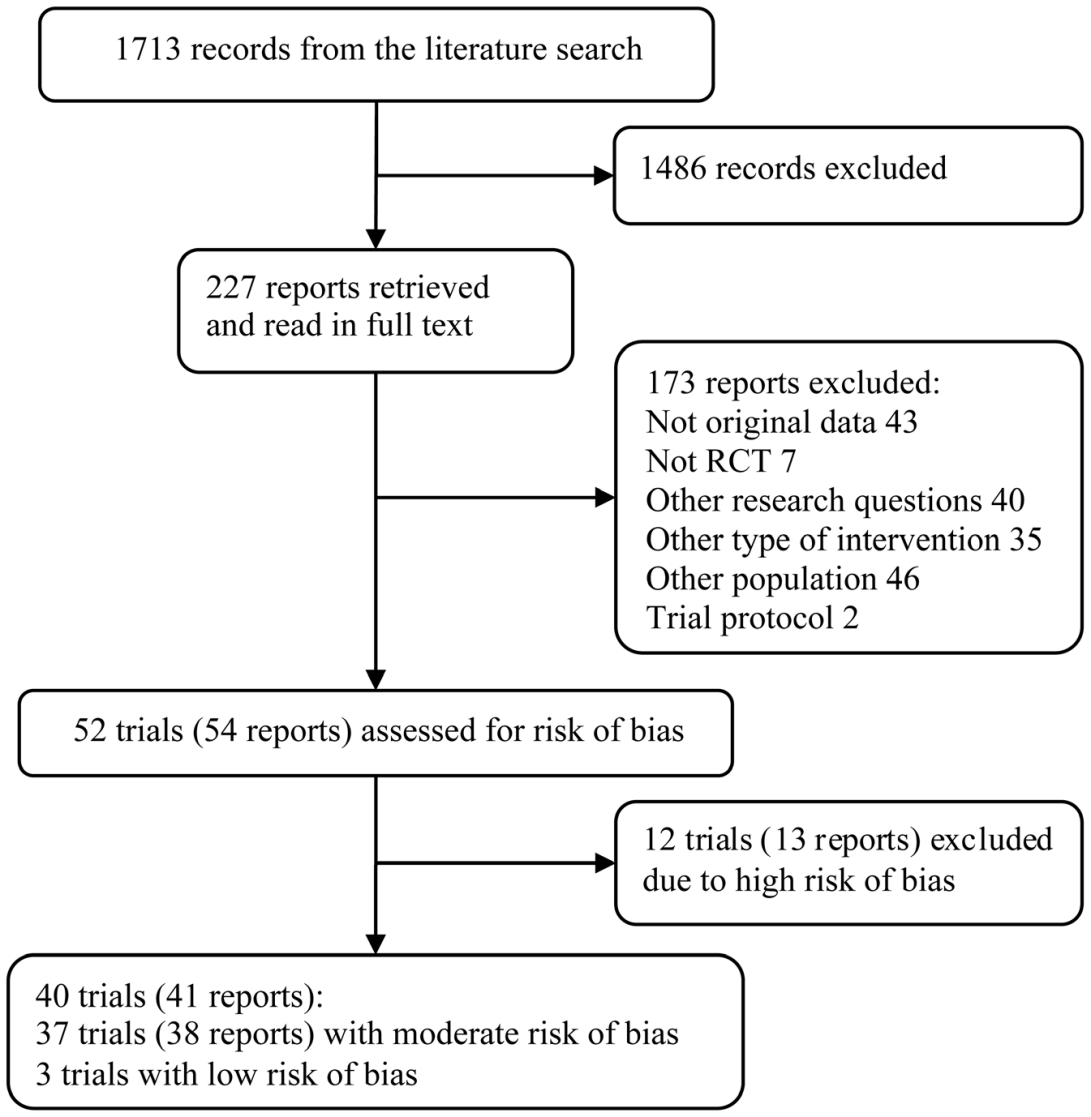
Flowchart of included efficacy trials and additional reports of long-term follow-up assessments.

We identified 52 relevant trials (54 reports), whereof 12 trials (13 reports) were excluded due to high risk of bias. The efficacy data thus included 39 reports with 40 RCTs of low or moderate risk of bias and 2 additional reports of long-term follow-ups of these trials that were included in the synthesis (Figure 1). Most trials recruited volunteers via advertisements, evaluated variations of therapist-guided I-CBT in self-help format carried out over 8–12 weeks and used a WLC (Table 1). The support was delivered via phone or email-like messages and took approximately 10–20 minutes per participant and week. Diagnoses were made mainly by using the MINI neuropsychiatric interview or the Structured Clinical Interview for DSM Axis-I Disorders (SCID) and the screening was performed in person or via telephone. The majority of the trials (88%) were conducted by teams from Australia or Sweden.

**Table 1:** Randomized controlled trials assessed as having low or moderate risk of bias.

Diagnosis	Age,	Women	Employed	Married	Current	N	Intervention; support	Comparator
Study, year, country	M yrs		/student^a^	/de facto relationship	medication^b^			
*Depressive Disorders* c								
Berger, 2011, Switzerland [44]	40	70%	75%	24%	25%	76	I-CBT; clinical	Waiting list
							I-CBT; none	
Carlbring, 2013, Sweden [38]	44	83%	81%	55%	14%	80	ACT, BA, mindfulness; clinical	Waiting list
Choi, 2012, Australia [42]	39	80%	66%	56%	18%	63	I-CBT; clinical	Waiting list
Johansson, 2012a, Sweden [40]	45	71%	74%	49%	31%	121	I-CBT, tailored; clinical	Online forum
							I-CBT, standardized; clinical	
Johansson, 2012b, Sweden [31]	46	75%	76%	65%	25%	92	I-PDT; clinical	Online supportive therapy
Kessler, 2009, England [43]						297	Therapist-led CBT via chat	TAU
Titov, 2010, Australia [41]	43	74%	73%	47%	58%	141	I-CBT; clinical	Waiting list
							I-CBT; nonclinical	
Vernmark, 2010, Sweden [39]	37	68%	72%	51%	19%	88	I-CBT; clinical	Waiting list
							Therapist-led CBT via email	
*Social Phobia*								
Andersson 2006, Sweden [49]	37	52%	91%	66%	22%	64	I-CBT; clinical	Waiting list
Andersson 2012, Sweden [54]	38	60%	89%	65%	14%	204	I-CBT; clinical	Online forum
Berger 2009, Switzerland [50]	29	56%	–	42%	–	52	I-CBT; clinical	Waiting list
Boettcher 2012, Germany/Switzerland [48]	38	37%	–	65%	6%	68	I-ABM	Sham therapy
Carlbring 2007, Sweden [55]	33	65%	–	58%	–	60	I-CBT; clinical	Waiting list
Carlbring 2012, Sweden [46]	37	68%	–	56%	22%	79	I-ABM	Sham therapy
Furmark 2009a, Sweden [56]	35	71%	80%	59%	8%	80	I-CBT; clinical	Waiting list
Furmark 2009b, Sweden [56]	34	66%	88%	52%	14%	58	I-CBT; clinical	Bibliotherapy
Hedman 2011, Sweden [47]	35	36%	74%	–	25%	126	I-CBT; clinical	Group CBT
Neubauer 2013, Germany [57]	40	66%	–	–	7%	59	I-ABM	Sham therapy
Titov 2008a, Australia [51]	38	59%	83%	46%	30%	105	I-CBT; clinical	Waiting list
Titov 2008b, Australia [52]	37	63%	90%	54%	26%	88	I-CBT; clinical	Waiting list
Titov 2008c, Australia [53]	38	61%	90%	52%	22%	98	I-CBT; clinical	Waiting list
							I-CBT; none	
*Panic Disorder*								
Bergström 2010, Sweden [60]	34	62%	77%	–	45%	113	I-CBT; clinical	Group CBT
Carlbring 2005, Sweden [63]	35	71%	–	–	51%	49	I-CBT; clinical	Individual CBT
Carlbring 2006, Sweden [62]	37	60%	75%	67%	54%	60	I-CBT; clinical	Waiting list
Klein 2006, Australia [64]	(18–70)	80%	–	–	53%	37	I-CBT; clinical	Waiting list
Wims 2010, Australia [61]	42	76%	83%	57%	39%	59	I-CBT; clinical	Waiting list
*GAD*								
Andersson 2012, Sweden [65]	40	77%	62%	67%	32%	81	I-PDT; clinical	Waiting list
							I-CBT; clinical	
Paxling 2011, Sweden [66]	39	80%	–	–	37%	89	I-CBT; clinical	Waiting list
Robinson 2010, Australia [67]	47	68%	76%	64%	32%	150	I-CBT; clinical	Waiting list
							I-CBT; nonclinical	
Titov 2009, Australia [68]	44	76%	40%	67%	29%	48	I-CBT; clinical	Waiting list
*OCD*								
Andersson 2012, Sweden [71]	34	66%	93%	–	23%	101	I-CBT; clinical	Online supportive therapy
*PTSD*								
Spence 2011, Australia [70]	43	81%	60%	45%	57%	44	I-CBT; clinical	Waiting list
*Specific Phobia* (spider phobia)								
Andersson 2009, Sweden [69]	26	85%	–	–	–	30	I-CBT; clinical	Individual CBT
*Various Anxiety Disorders* d								
Bell 2012, Australia [75]	35	67%	69%	–	60%	83	I-CBT; nonclinical	Waiting list
Carlbring 2011, Sweden [74]	39	76%	76%	67%	26%	54	I-CBT; clinical	Waiting list
Johnston 2011, Australia [72]	42	59%	84%	50%	29%	139	I-CBT; clinical	Waiting list
							I-CBT; nonclinical	
Newby 2013, Australia [77]	44	78%	66%	63%	40%	109	I-CBT; clinical	Waiting list
Titov 2010, Australia [73]	40	68%	82%	53%	47%	86	I-CBT; clinical	Waiting list
Titov 2011, Australia [76]	44	73%	63%	46%	54%	77	I-CBT; clinical	Waiting list
March 2009, Australia [78]	9	55%	–	–	0%	72	I-CBT; clinical	Waiting list

ABM =  Attention bias modification. CBT =  Cognitive behavior therapy. GAD =  Generalized anxiety disorder. OCD =  Obsessive-compulsive disorder. PD =  Panic disorder. PDT =  Psychodynamic therapy. PTSD =  Posttraumatic stress disorder.

a Includes full- and part-time employed and students. For a few studies that only reported number of participants on sick leave, we report the proportion not on sick leave.

b Psychotropic medication, although this was not stated explicitly in each report.

c Includes major depressive episode acute/in partial remission, major depressive disorder, dysthymia; mainly mild/moderate severity, low suicidality.

d Mainly GAD, social anxiety disorder, panic disorder; also major depressive disorder (*k* = 2).

### Mood disorders in adults

Nine trials were identified: eight had moderate risk of bias [31], [38]–[44] and one had high risk of bias (Appendix S2). The participants fulfilled criteria for a depressive episode, current or in partial remission, recurrent episodes, or dysthymia. No trials for bipolar disorder were found. Six trials included only participants with mild to moderate depression and six trials excluded participants who reported suicidal ideation.

**Figure 2:**
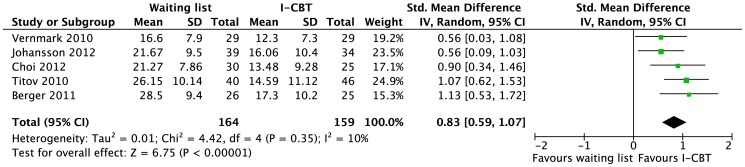
Short-term efficacy of therapist-guided internet-based cognitive behavioral therapy (I-CBT) vs. waiting list for depression in adults. For the meta-analysis, the outcome chosen from each study was the Beck Depression Inventory I or II.

None of the trials assessed noninferiority. Five evaluated the effect of I-CBT vs. a WLC [41], [42], [44], WLC and weekly symptom ratings [39], or WLC and access to an online discussion group [40]. We found a large pooled effect for I-CBT as compared to a WLC (Figure 2). The quality of evidence was rated as moderate for therapist-guided I-CBT due to study limitations (WLC), see Table 2.

**Table 2:** Evidence of Short-term Efficacy of Internet-Delivered Psychological Treatments for Mood and Anxiety Disorders.

Disorder	Treatment	Support	Comparator	Outcome	Results	*N*/*k*	Quality of evidence (GRADE)
*Adults*							
Unipolar depression	CBT	Clinical	Waiting list	BDI	CBT> waiting list	323/5	⊕⊕⊕○^a^
	CBT	None	Waiting list	BDI	CBT> waiting list	51/1	⊕○○○^a^^,^ b ^,^ c
	PDT	Clinical	Online supportive therapy	BDI	n.s.	92/1	⊕○○○^a^^,^ b ^,^ c
	Chat CBT	–	Usual GP care	BDI	CBT> usual GP care	297/1	⊕○○○^a^^,^ b ^,^ c
	ACT	Clinical	Waiting list	BDI	n.s.	80/1	⊕○○○^a^^,^ b ^,^ c
Social phobia	CBT	Clinical	Live group CBT	LSAS, SPS, SIAS	Noninferior	126/1	⊕⊕○○^b^^,^ c
	CBT	Clinical	Waiting list	SPS, SIAS	CBT> waiting list	709/8	⊕⊕⊕○^a^
	CBT	Clinical	Bibliotherapy	LSAS, SPS, SIAS	n.s.	58/1	⊕○○○^b^^,^ c
	ABM	–	Sham therapy	LSAS, SPS, SIAS	n.s.	206/3	⊕⊕⊕○^b^
	CBT	None	Waiting list	SPS, SIAS	n.s.	66/1	⊕○○○^a^^,^ b ^,^ c
Panic disorder	CBT	Clinical	Waiting list	Varied among studies	CBT> waiting list	151/3	⊕⊕○○^a^^,^ b
	CBT	Clinical	Individual CBT	BSQ	n.s.	49/1	⊕○○○^a^^,^ b ^,^ c
	CBT	Clinical	Group CBT	PDSS	n.s.	93/1	⊕○○○^a^^,^ b ^,^ c
GAD	CBT	Clinical	Waiting list	PSWQ	CBT> waiting list	271/4	⊕⊕○○^a^^,^ b
	CBT	Non-clinical	Waiting list	PSWQ	CBT> waiting list	99/1	⊕○○○^a^^,^ b ^,^ c
	PDT	Clinical	Waiting list	PSWQ	n.s.	54/1	⊕○○○^a^^,^ b ^,^ c
Specific phobia	CBT	Clinical	Live CBT	BAT	n.s.	30/1	⊕○○○^a^^,^ b ^,^ c
OCD	CBT	Clinical	Online supportive therapy	Y-BOCS	CBT> support	101/1	⊕○○○^a^^,^ b ^,^ c
PTSD	CBT	Clinical	Waiting list	PCL-C	CBT> waiting list	44/1	⊕○○○^a^^,^ b ^,^ c
Anxiety disorders	CBT	Clinical	Waiting list	Varied among studies	CBT> waiting list	414/5	⊕⊕○○^a^^,^ b
Anxiety disorders	CBT	Non-clinical	Waiting list	WSAS, LSAS, PSWQ, GAI, FQ	n.s.	83/1	⊕○○○^a^^,^ b ^,^ c
*Children*							
Anxiety disorders	CBT	Clinical	Waiting list	ADIS	CBT> waiting list	72/1	⊕○○○^a^^,^ b ^,^ c

ABM =  Attention bias modification. ADIS =  Anxiety Disorders Interview Schedule. BAT =  Behavioral approach test. BDI =  Beck Depression Inventory (I or II). BSQ =  Body Sensations Questionnaire. CBT =  Cognitive behavior therapy. FQ =  Fear Questionnaire. GAD =  Generalized anxiety disorder. GAI =  Generalized Anxiety Inventory. LSAS =  Liebowitz Social Anxiety Scale. OCD =  Obsessive-compulsive disorder. PCL-C =  PTSD Checklist–Civilian version. PDSS =  Panic Disorder Severity Scale, Self-Report. PDT =  Psychodynamic therapy. PSWQ =  Penn State Worry Questionnaire. SIAS =  Social Interaction Anxiety Scale. PTSD =  Posttraumatic stress disorder. SPS =  Social Phobia Scale. Y-BOCS =  Yale-Brown Obsessive-Compulsive Scale.

a Study limitations (risk of bias);

b Imprecision (e.g., small samples, heterogeneous effect sizes);

c Indirectness (e.g., single trial).

Three other trials were included that evaluated one intervention each: one trial with an intervention that combined components from acceptance and commitment therapy, behavioral activation, and mindfulness [38]; one with internet-delivered psychodynamic therapy (I-PDT) [31], and one with therapist-led I-CBT delivered via a chat interface [43]. For each intervention, the quality of evidence was rated as very low. Four long-term follow-up assessments (five reports) were assessed as having a high risk of bias [31], [39], [40], [44], [45].

### Anxiety disorders in adults

#### Social phobia

Sixteen trials were identified: three trials had low risk of bias [46]–[48], 10 trials reported in 9 publications had moderate risk [49]–[57], and 3 trials had high risk. One noninferiority trial with low risk of bias found that therapist-guided I-CBT was superior to live group CBT, with an effect size of *d* = 0.41 on the LSAS (blinded) [47]. The 95% CI (0.03 to 0.78) was above our pre-defined noninferiority margin. We rated the quality of evidence as low because of imprecision (sample size) and indirectness (single trial).

**Figure 3:**
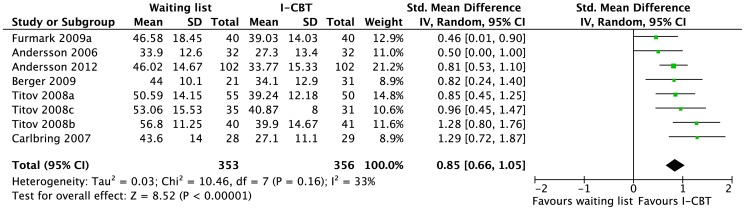
Short-term efficacy of therapist-guided internet-based cognitive behavioral therapy (I-CBT) vs. waiting list for social phobia in adults. For the meta-analysis, the outcome chosen from each study was the Social Interaction Anxiety Scale.

Eight trials with moderate risk of bias evaluated the effect of therapist-guided I-CBT compared to a WLC [49]–[56]. The treatments conferred a large effect compared to WLC (Figure 3). The quality of evidence for therapist-guided I-CBT was rated as moderate due to study limitations (WLC). One report also evaluated whether therapist-guided I-CBT was superior to bibliotherapy [56]. I-CBT was not found to be superior to bibliotherapy. The quality of evidence for guided I-CBT vs. bibliotherapy was rated as very low due to imprecision (small sample) and indirectness (single trial).

Two trials (three reports) [56], [58], [59] included long-term follow-ups of the treatment groups were assessed as having moderate risk of bias. Their findings suggested that participants' improvements persisted after 30 months [58] and 1 and 5 years [56], [59]. The quality of evidence was assessed as very low due to risk of bias and imprecision. One trial found that unguided I-CBT was not superior to a WLC [53]. The quality of evidence for unguided I-CBT for social phobia was rated as very low due to study limitations (WLC), imprecision (small sample), and indirectness (single trial).

**Figure 4:**
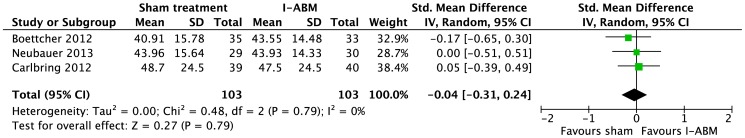
Short-term efficacy of internet-based attention bias modification (I-ABM) vs. sham treatment for social phobia in adults. For the meta-analysis, the outcome chosen from each study was the Social Interaction Anxiety Scale.

Three trials, two of low [46], [48] and one of moderate risk of bias [57], compared internet-delivered Attention Bias Modification (I-ABM) to an identical sham intervention. We found no clinically relevant pooled effect (Figure 4 includes one of three primary outcomes; plots were nearly identical for the Social Phobia Scale and the Liebowitz Social Anxiety Scale). The quality of evidence was rated as moderate for a lack of clinically meaningful effect of I-ABM (rated down due to imprecision, i.e., small sample).

#### Panic disorder

Nine trials of I-CBT were identified. Five trials had moderate risk [60]–[64] and four had high risk of bias (e.g., due to differences among groups in baseline characteristics, sample sizes, dropout). One trial found no difference between therapist-guided I-CBT and live group CBT in participants recruited from a clinical population (*d* = 0.00) [60]. Noninferiority was not established as the 95% CI (−0.41 to 0.41) included our predefined noninferiority margin of *d* = −0.20. One trial found no difference between I-CBT and live individual CBT [63]. This trial was not designed as a noninferiority trial and the small sample limits the inferences to be made. The quality of evidence for the noninferiority of I-CBT vs. either individual or group CBT thus was rated as very low due to study limitations (e.g., insufficient information about treatment integrity), imprecision (small sample), and indirectness (single trial).

Three trials that compared therapist-guided I-CBT vs. a WLC with [64] or without [61], [62] online information about panic found small to very large effects. No meta-analysis was undertaken because of the heterogeneity in outcome measures and effect sizes. We rated the quality of evidence as low because of study limitations (WLC, dropout) and imprecision (heterogeneous effect sizes, small samples).

#### Generalized anxiety disorder

**Figure 5:**
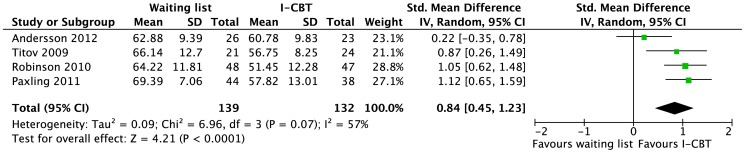
Short-term efficacy of internet-based cognitive behavioral therapy (I-CBT) vs. waiting list for generalized anxiety disorder in adults. For the meta-analysis, the outcome chosen from each study was the Penn State Worry Questionnaire.

Four trials with moderate risk of bias were identified [65]–[68]. They evaluated therapist-guided I-CBT vs. a WLC. The pooled effect was large although heterogeneous across the trials (Figure 5). We rated the quality of evidence as low for the short-term effect because of study limitations (WLC) and imprecision (heterogeneous effect sizes, small samples).

One trial found that I-CBT with non-clinical support by a technician was more effective than a WLC [67]. One trial included therapist-guided I-PDT [65]. As for the I-CBT condition in this trial no effect was found for I-PDT vs. WLC. The quality of evidence for I-CBT with non-clinical support and therapist-guided I-PDT was rated as very low because of study limitations (WLC, only one technician), imprecision (small sample), and indirectness (single trial).

#### Specific phobia

We identified one trial of moderate risk of bias [69]. Four weeks of therapist-guided I-CBT did not outperform brief therapist-led exposure (one introductory session and one three-hour exposure session) according to a behavioral approach test in participants with spider phobia. The quality of evidence was rated as very low due to study limitations, imprecision (small sample), and indirectness (single trial).

#### Posttraumatic stress disorder

Two relevant trials were identified; one trial with high risk of bias and one trial with moderate risk of bias that found that therapist-guided I-CBT was superior to WLC [70]. The quality of evidence was rated as very low due to study limitations (WLC), imprecision (small sample), and indirectness (single trial).

#### Obsessive-compulsive disorder

We identified one trial of moderate risk of bias [71]. Therapist-guided I-CBT conferred a large effect compared to supportive therapy online. The quality of evidence was rated as very low due to study limitations (no credible active comparison condition), imprecision (small sample), and indirectness (single trial).

#### Transdiagnostic interventions for anxiety and depression

Six trials of moderate risk of bias were identified that included participants with mixed anxiety disorders and/or MDD [72]–[77]. Five trials found that therapist-guided I-CBT had moderate or large effects as compared to a WLC [72]–[74], [76]. No meta-analysis was performed due to the heterogeneity in outcome measures, diagnoses, and treatment protocols. We rated the quality of evidence as low for these interventions because of study limitations (WLC) and imprecision (small samples, heterogeneous effects and interventions). One trial included a 1- and 2-year follow-up, with results suggesting that the improvements lasted throughout the follow up [74]. The quality of evidence was rated as very low due to study limitations (observational design, dropout) and imprecision (small sample). One trial recruited participants from an anxiety clinic and found no difference between unguided I-CBT and a WLC on the Patient Global Impression scale [75]. The quality of evidence was rated as very low due to study limitations (WLC, dropout), imprecision (small sample), and indirectness (single trial).

### Publication bias

Funnel plots and Duval and Tweedie's trim-and-fill procedure indicated no or trivial publication bias with respect to the pooled effect sizes for I-CBT for adults with depression, social phobia, and GAD.

### Children and adolescents

We found four trials and excluded three due to high risk of bias because of various shortcomings. One trial of moderate risk of bias evaluated I-CBT for mixed anxiety disorders [78]: 30% of the completers did no longer fulfill criteria for their primary anxiety diagnosis, compared to 10% in the WLC. The quality of evidence was rated as very low for the efficacy of internet-based psychological interventions for children and adolescents (Table 2).

### Risk of adverse events

Eight trials provided information on intervention-associated risks for depression [31], [38], social phobia [46], [54], GAD [65], OCD [71], and transdiagnostic treatments [74], [76]. The information provided was related to a worsening in symptoms and indicated that symptom worsening was present in 0–5% of treated participants and in 2–9% of participants in the comparison groups. The quality of evidence was rated as very low for the risk of adverse events following internet-based psychological interventions for both children and adults (⊕○○○).

### Cost-effectiveness

Of the 139 studies screened for cost-effectiveness data, five trials met the eligibility criteria. Two had a moderate risk of bias [79], [80] and three were excluded due to high risk of bias (e.g. incomplete information on costs; Appendix S2). One trial compared costs and effects between I-CBT and treatment as usual while on waiting list among patients with depression [80], and found that I-CBT had a cost per QALY of 29,384 USD compared to treatment as usual. At a willingness-to-pay for a QALY of 50,000 USD the probability was approximately 70% that I-CBT was cost-effective compared to treatment as usual. One trial compared costs and effects between I-CBT and group CBT among patients with social phobia [79]. Compared to group CBT, I-CBT was associated with a lower cost per patient of 1,422 USD and 19% greater improvement on LSAS at the six-month follow-up. At a willingness-to-pay per additionally improved patient of 3,000 USD, the probability that I-CBT was cost-effective compared to group CBT was approximately 90%. The calculations of QALYs had not taken the time aspect of the effect on quality of life into account and are not presented.

## Discussion

In this review we assessed whether internet-delivered psychological treatments for mood and anxiety disorders are efficacious, noninferior to established treatments, associated with risk of adverse events, and cost-effective. We found limited to moderate evidence that for adults who seek out this treatment, therapist-guided I-CBT has a favorable short-term effect compared to waiting list for social phobia, panic disorder, generalized anxiety disorder, or mild to moderate major depression. We were not able to draw conclusions about noninferiority to proven treatments, long-term effects, adverse events, cost-effectiveness, or efficacy when given to children and adolescents.

Several reviews interpret the body of evidence such that I-CBT and established forms of CBT have comparable effects for mild to moderate depression and several anxiety disorders [19], [20], [81]. In contrast, we found insufficient evidence to conclude whether I-CBT is noninferior to face-to-face treatment. There are important aspects that need to be attended to with regard to the methodology, and ethics, of conducting trials with a placebo/no-treatment arm when there are existing evidence-based treatments [82], [83]. These issues notwithstanding, we found few trials that compared I-CBT to a face-to-face treatment. These trials were generally not adequately designed to evaluate questions of noninferiority [27], [84], with an exception of one trial, which provided tentative support for similar efficacy of therapist-guided I-CBT and group CBT for social phobia in adults [47].

There are a number of shortcomings with the existing trials that future studies would benefit from attending to. A common issue to these studies is that they were conducted by teams that developed the I-CBT program but had no role in developing the comparison face-to-face therapy. In addition, independent ratings of the quality of delivery of the therapy were not routinely included. Further, the face-to-face comparator was often group CBT and not individual CBT although the latter is generally the first-hand choice for anxiety and mood disorders [85], [86]. The guidelines from the National Institute for Health and Care Excellence (NICE) do not support the notion of equivalence between internet-delivered and face-to-face treatment for social phobia [86], in part due to the aforementioned issues. More aptly designed trials are needed before we can answer clearly whether internet-delivered treatments are noninferior to face-to-face treatment. Furthermore, the lack of comparisons with established treatments provide scant data for cost-effectiveness analyses. Consequently, this review can provide no conclusions about the cost-effectiveness of I-CBT.

The diverging conclusions among extant reviews about equal efficacy between internet-delivered and face-to-face treatments highlight critical methodological aspects that set the present review apart from previous reviews [19], [81], [87]. First, we used rigorous criteria for establishing noninferiority, whereas previous reviews seemingly have relied on subjective and indirect appraisal of the effect size differences. Second, we performed a systematic assessment of the body of evidence for each disorder [23] whereas previous reviews either used no formal assessment or relied only on the criteria stated by Chambless et al. [88], which indicate as evidence-based treatment any treatment that have been found superior to any comparison condition in two RCTs. The grading of the body of evidence that we used here entailed a reduced confidence in the results mainly due to the fact that studies were unblinded, used subjective outcome measures, were designed with waiting list or similar comparison groups, and included relatively small samples.

Third, we performed a comprehensive assessment of the risk of bias in the trials and excluded trials with high risk. Few trials for social phobia and depression were judged as having high risk of bias, which resulted in similar conclusions about short-term efficacy as the meta-analysis by Andrews et al. [19] Similarly, for PTSD, OCD, specific phobia, and transdiagnostic treatments only one trial (for PTSD) was excluded due to high risk. However, excluding high-risk studies resulted in fewer trials and a lower grading of the evidence for panic disorder than stated in previous reviews [19], [89]. Of the four excluded publications on panic disorder two publications were from 2001, one from 2006, and one from 2008 (see Appendix S2). Given the technical progress in the field and that the reports represent studies planned and performed some years before publication, at least the 2001 publications are among the first in an emerging field and would have less resemblance of current and future practice of internet-delivered treatment packages.

We found only four relevant trials for children and adolescents, and three had high risk of bias. The three excluded trials concerned social anxiety, OCD, and diverse anxiety disorders (mainly GAD), respectively. Including them would not alter our conclusions. This turnout seems to reflect the slow progress in general among psychological interventions for children and adolescents [90]. Although the low number of studies precludes quantitative meta-analysis, an equally important objective of a systematic review is to identify gaps in the literature. This could alert researchers and funding agencies to important research questions that are not given sufficient attention. The effect of internet-delivered interventions in general may be smaller among children [91], which stresses the need for more research specifically for this population.

Finally, the trials included in this review may seem few in comparison to the expanding number of publications in the literature. However, we only included studies of participants with diagnosed mood and anxiety disorders. There are many other studies on internet-treatments in which participants have not been subjected to a diagnostic interview. Several of those trials used unguided interventions, which may explain why so few trials of unguided interventions were included. Also, we did not pool studies across treatments and support types, or across disorders, and therefore each cluster of studies yielded a modest number of trials despite an impressive amount overall.

Remote delivery is one of several promising avenues for expanding the reach of psychological interventions [5]. Indeed, a key impetus in much of the reviewed research is to improve accessibility to CBT [63] and attract those normally too shy to seek treatment and those without access to CBT [92]. A central question, therefore, is whether internet-delivered treatment indeed attracts an underserved population. Among the trials of I-CBT for depression, 53–61% of participants had a history of psychological treatment [39], [40], [44]. Among the anxiety trials 16–66% of participants had previously received psychological treatment [49]–[52], [54], [56], [71] and one-fourth had received CBT [63], [71]. The data indicate that many depression trial participants already had access to treatment whereas this seemed to be less clear for anxiety disorders. The high level of educational attainment and employment rates among the participants raise concerns about whether the effects found in most RCTs can be generalized to those who today are underserved. Other questions also likely to be important to generalization concerns how these treatment programs can be implemented within the healthcare services and what type of changes that would be needed; for example, the training of existing therapists. Expanding the reach of psychological treatments is important [5]. We therefore concur with the NICE guidance [86] and hope for further research that attend to these issues in more detail.

Several trials assessed long-term outcomes of the treatments. Yet, no clear conclusions could be drawn about long-term effects as these data were limited mainly due to the observational design, attrition, and the lack of data on participants' receipt of other treatments during the follow-up period. Only eight efficacy trials reported on deterioration, and no trial suggested that adverse events in a broader sense had been monitored. There is clearly a need for better reporting of risk of safety data [93], [94]. Currently, the risk of reporting bias precludes conclusions about the risk-benefit ratio of the treatments, which is an important aspect of comparing treatments.

Correlational evidence suggests that therapist guidance is beneficial for the outcome [16], [95], [96]. Less extensive support without adequate oversight of the patients' mental health status could also compromise patient safety. We therefore emphasize that evidence was found only for therapist-guided treatments. The lack of efficacy of I-ABM (also seen in a trial published after our final search [97]) compared to the effects of ABM in the laboratory [98], and of other remotely delivered therapies [99] further indicates the importance of attention to details about how interventions are delivered.

We believe that using only trials with low or moderate risk of bias is an improvement to previous reviews. We are mindful of the fact that the ratings of risk of bias were subjective, which hampers the replicability of our findings. However, it is broadly recognized as poor review practice to disregard study quality altogether [26], for example, because of the impact of quality on effect estimates [28]. Instead of choosing a threshold approach, a quality-weighting approach can be used whereby low quality studies are included in the review and their influence is analyzed, thus avoiding selection bias. However, the assignment of quality weights is still fraught with subjectivity: unless rigorously implemented, it might increase the risk of over-inclusion bias and may result in inconsistency [100]. In addition, the use of simple scoring sheets for assessing bias is not recommended [26]. To minimize the uncertainty due to subjective judgments we performed the ratings according to best practice: The risk of conflicts of interests were minimized by the choice of independent reviewers and we used comprehensive score sheets developed for risk of bias ratings in individual trials and dual review; and we used the GRADE model for the overall assessment of the evidence [23]. Our ratings of the strength of evidence are related not only to specific treatment packages and comparison conditions (Table 2), but also to the particular population of adults seeking out treatment themselves. The majority of the included trials were conducted in Sweden or Australia, which greatly increases external validity within these countries; however, also warranting caution before extrapolating these findings into healthcare services in other countries and cultural settings.

### Conclusions

I-CBT for adults with mild to moderate depression and select anxiety disorders may complement existing services. More research is needed before conclusions can be drawn about the efficacy of internet-delivered treatment regarding other anxiety disorders, other treatment methods than CBT, the treatment of children, long-term effects, safety, cost-effectiveness, and noninferiority to proven forms of treatment. We believe that a shift is warranted from waiting list trials to using active comparators, particularly direct comparisons with established treatments. Nonetheless, more research is needed to understand what makes psychological treatments effective, and for whom. This field unfolds rapidly, however, and it may not be long until remaining questions can be satisfactorily answered.

## Supporting Information

Appendix S1 **Detailed search strategy.**
(DOCX)

Appendix S2 **List of reports excluded after full-text reading.**
(DOCX)

Checklist S1 **PRISMA checklist.**
(DOC)

Protocol S1 **Study protocol.**
(DOCX)

## References

[1] Wittchen HU, Jacobi F, Rehm J, Gustavsson A, Svensson M, et al (2011) The size and burden of mental disorders and other disorders of the brain in Europe 2010. Eur Neuropsychopharmacol 21: 655–679. 21896369 10.1016/j.euroneuro.2011.07.018

[2] Kessler RC, Chiu WT, Demler O, Walters EE (2005) Prevalence, severity, and comorbidity of 12-month DSM-IV disorders in the National Comorbidity Survey Replication. Arch Gen Psychiatry 62: 617–627. 15939839 10.1001/archpsyc.62.6.617 PMC2847357

[3] Lépine JP, Briley M (2011) The increasing burden of depression. Neuropsychiatr Dis Treat 7: 3–7. 21750622 10.2147/NDT.S19617 PMC3131101

[4] Kessler RC, Ruscio AM, Shear K, Wittchen HU (2010) Epidemiology of anxiety disorders. In: Stein MB, Steckler T, editors. Behavioral neurobiology of anxiety and its treatment. New York: Springer. pp. 21–35. 21309104

[5] Kazdin AE, Blase SL (2011) Rebooting psychotherapy research and practice to reduce the burden of mental illness. Perspect Psychol Sci 6: 21–37. 26162113 10.1177/1745691610393527

[6] Trivedi MH, Rush AJ, Wisniewski SR, Nierenberg AA, Warden D, et al (2006) Evaluation of outcomes with citalopram for depression using measurement-based care in STAR*D: implications for clinical practice. Am J Psychiatry 163: 28–40. 16390886 10.1176/appi.ajp.163.1.28

[7] McHugh RK, Whitton SW, Peckham AD, Welge JA, Otto MW (2013) Patient preference for psychological vs pharmacologic treatment of psychiatric disorders: a meta-analytic review. J Clin Psychiatry 74: 595–602. 23842011 10.4088/JCP.12r07757 PMC4156137

[8] Hofmann SG, Smits JA (2008) Cognitive-behavioral therapy for adult anxiety disorders: a meta-analysis of randomized placebo-controlled trials. J Clin Psychiatry 69: 621–632. 18363421 10.4088/jcp.v69n0415 PMC2409267

[9] Butler AC, Chapman JE, Forman EM, Beck AT (2006) The empirical status of cognitive-behavioral therapy: a review of meta-analyses. Clin Psychol Rev 26: 17–31. 16199119 10.1016/j.cpr.2005.07.003

[10] Tolin DF (2010) Is cognitive–behavioral therapy more effective than other therapies? A meta-analytic review. Clin Psychol Rev 30: 710–720. 20547435 10.1016/j.cpr.2010.05.003

[11] James AC, James G, Cowdrey FA, Soler A, Choke A (2013) Cognitive behavioural therapy for anxiety disorders in children and adolescents. Cochrane Database Syst Rev 6: CD004690. 10.1002/14651858.CD004690.pub3 23733328

[12] Cuijpers P, Geraedts AS, van Oppen P, Andersson G, Markowitz JC, et al (2011) Interpersonal psychotherapy for depression: a meta-analysis. Am J Psychiatry 168: 581–592. 21362740 10.1176/appi.ajp.2010.10101411 PMC3646065

[13] Shedler J (2010) The efficacy of psychodynamic psychotherapy. Am Psychol 65: 98–109. 20141265 10.1037/a0018378

[14] Demyttenaere K, Bruffaerts R, Posada-Villa J, Gasquet I, Kovess V, et al (2004) Prevalence, severity, and unmet need for treatment of mental disorders in the World Health Organization World Mental Health Surveys. JAMA 291: 2581–2590. 15173149 10.1001/jama.291.21.2581

[15] Stein MB (2004) Quality of care for primary care patients with anxiety disorders. Am J Psychiatry 161: 2230–2237. 15569894 10.1176/appi.ajp.161.12.2230

[16] Spek V, Cuijpers P, Nyklicek I, Riper H, Keyzer J, et al (2007) Internet-based cognitive behaviour therapy for symptoms of depression and anxiety: a meta-analysis. Psychol Med 37: 319–328. 17112400 10.1017/S0033291706008944

[17] Griffiths KM, Farrer L, Christensen H (2010) The efficacy of internet interventions for depression and anxiety disorders: a review of randomised controlled trials. Med J Aust 192: S4–11. 20528707 10.5694/j.1326-5377.2010.tb03685.x

[18] Andersson G (2009) Using the Internet to provide cognitive behaviour therapy. Behav Res Ther 47: 175–180. 19230862 10.1016/j.brat.2009.01.010

[19] Andrews G, Cuijpers P, Craske MG, McEvoy P, Titov N (2010) Computer therapy for the anxiety and depressive disorders is effective, acceptable and practical health care: a meta-analysis. PLoS One 5: e13196. 20967242 10.1371/journal.pone.0013196 PMC2954140

[20] Hedman E, Ljotsson B, Lindefors N (2012) Cognitive behavior therapy via the Internet: a systematic review of applications, clinical efficacy and cost-effectiveness. Expert Rev Pharmacoecon Outcomes Res 12: 745–764. 23252357 10.1586/erp.12.67

[21] Guyatt GH, Oxman AD, Vist G, Kunz R, Brozek J, et al (2011) GRADE guidelines: 4. Rating the quality of evidence–study limitations (risk of bias). J Clin Epidemiol 64: 407–415. 21247734 10.1016/j.jclinepi.2010.07.017

[22] Cooney GM, Dwan K, Greig CA, Lawlor DA, Rimer J, et al (2013) Exercise for depression. Cochrane Database Syst Rev 9: CD004366. 10.1002/14651858.CD004366.pub6 PMC9721454 24026850

[23] Balshem H, Helfand M, Schunemann HJ, Oxman AD, Kunz R, et al (2011) GRADE guidelines: 3. Rating the quality of evidence. J Clin Epidemiol 64: 401–406. 21208779 10.1016/j.jclinepi.2010.07.015

[24] Andersson G, Cuijpers P (2009) Internet-based and other computerized psychological treatments for adult depression: a meta-analysis. Cogn Behav Ther 38: 196–205. 20183695 10.1080/16506070903318960

[25] Collaboration TC (2014) Cochrane editorial and publishing policy resource: Conflicts of interest and Cochrane Reviews. Oxford, UK: The Cochrane Collaboration.

[26] Higgins J, Altman D, Sterne JAC (editors) (2011) Chapter 8: Assessing risk of bias in included studies. In: Higgins J, Green S, editors. Cochrane Handbook for Systematic Reviews of Interventions Version 510 (updated March 2011): The Cochrane Collaboration.

[27] Schumi J, Wittes JT (2011) Through the looking glass: understanding non-inferiority. Trials 12: 106. 21539749 10.1186/1745-6215-12-106 PMC3113981

[28] Moher D, Pham B, Jones A, Cook DJ, Jadad AR, et al (1998) Does quality of reports of randomised trials affect estimates of intervention efficacy reported in meta-analyses? Lancet 352: 609–613. 9746022 10.1016/S0140-6736(98)01085-X

[29] D'Agostino RB Sr, Massaro JM, Sullivan LM (2003) Non-inferiority trials: design concepts and issues - the encounters of academic consultants in statistics. Stat Med 22: 169–186. 12520555 10.1002/sim.1425

[30] International Conference on Harmonisation of Technical Requirements for Registration of Pharmaceuticals for Human Use (ICH) (2000) Choice of control group and related issues in clinical trials: ICH harmonised tripartite guideline. Report nr E10. Geneva: ICH. Cited 23 Nov 2013. Available from: www.ich.org

[31] Johansson R, Ekbladh S, Hebert A, Lindström M, Möller S, et al (2012) Psychodynamic guided self-help for adult depression through the internet: a randomised controlled trial. PLoS One 7: e38021. 22741027 10.1371/journal.pone.0038021 PMC3362510

[32] American Psychiatric Association (1994) Diagnostic and statistical manual of mental disorders. 4th edition. Washington, DC: Author.

[33] World Health Organization (1992) ICD-10: International statistical classification of diseases and related health problems. 10th edition. Geneva: Author.

[34] Swedish Council on Health Technology Assessment (SBU) (2013) Utvärdering av metoder i hälso- och sjukvården: En handbok. [Evaluation and synthesis of methods in healthcare: A handbook]. Stockholm, Sweden: SBU Swedish.

[35] Cohen J (1988) Statistical power analysis for the behavioral sciences. 2nd edition. Hillsdale, NJ: Erlbaum. 567 p.

[36] Shemilt I, Thomas J, Morciano M (2010) A web-based tool for adjusting costs to a specific target currency and price year. Evid Policy 6: 51–59.

[37] Duval S, Tweedie R (2000) Trim and fill: a simple funnel-plot-based method of testing and adjusting for publication bias in meta-analysis. Biometrics 56: 455–463. 10877304 10.1111/j.0006-341x.2000.00455.x

[38] Carlbring P, Hagglund M, Luthstrom A, Dahlin M, Kadowaki A, et al (2013) Internet-based behavioral activation and acceptance-based treatment for depression: a randomized controlled trial. J Affect Disord 148: 331–337. 23357657 10.1016/j.jad.2012.12.020

[39] Vernmark K, Lenndin J, Bjarehed J, Carlsson M, Karlsson J, et al (2010) Internet administered guided self-help versus individualized e-mail therapy: a randomized trial of two versions of CBT for major depression. Behav Res Ther 48: 368–376. 20152960 10.1016/j.brat.2010.01.005

[40] Johansson R, Sjoberg E, Sjogren M, Johnsson E, Carlbring P, et al (2012) Tailored vs. standardized internet-based cognitive behavior therapy for depression and comorbid symptoms: a randomized controlled trial. PLoS One 7: e36905. 22615841 10.1371/journal.pone.0036905 PMC3352859

[41] Titov N, Andrews G, Davies M, McIntyre K, Robinson E, et al (2010) Internet treatment for depression: a randomized controlled trial comparing clinician vs. technician assistance. PLoS One 5: e10939. 20544030 10.1371/journal.pone.0010939 PMC2882336

[42] Choi I, Zou J, Titov N, Dear BF, Li S, et al (2012) Culturally attuned Internet treatment for depression amongst Chinese Australians: a randomised controlled trial. J Affect Disord 136: 459–468. 22177742 10.1016/j.jad.2011.11.003

[43] Kessler D, Lewis G, Kaur S, Wiles N, King M, et al (2009) Therapist-delivered Internet psychotherapy for depression in primary care: a randomised controlled trial. Lancet 374: 628–634. 19700005 10.1016/S0140-6736(09)61257-5

[44] Berger T, Hammerli K, Gubser N, Andersson G, Caspar F (2011) Internet-based treatment of depression: a randomized controlled trial comparing guided with unguided self-help. Cogn Behav Ther 40: 251–266. 22060248 10.1080/16506073.2011.616531

[45] Andersson G, Hesser H, Hummerdal D, Bergman-Nordgren L, Carlbring P (2013) A 3.5-year follow-up of Internet-delivered cognitive behavior therapy for major depression. J Ment Health 22: 155–164. 21957933 10.3109/09638237.2011.608747

[46] Carlbring P, Apelstrand M, Sehlin H, Amir N, Rousseau A, et al (2012) Internet-delivered attention bias modification training in individuals with social anxiety disorder: a double blind randomized controlled trial. BMC Psychiatry 12: 66. 22731889 10.1186/1471-244X-12-66 PMC3464865

[47] Hedman E, Andersson G, Ljotsson B, Andersson E, Ruck C, et al (2011) Internet-based cognitive behavior therapy vs. cognitive behavioral group therapy for social anxiety disorder: a randomized controlled non-inferiority trial. PLoS One 6: e18001. 21483704 10.1371/journal.pone.0018001 PMC3070741

[48] Boettcher J, Berger T, Renneberg B (2012) Internet-based attention training for social anxiety: a randomized controlled trial. Cognit Ther Res 36: 522–536.

[49] Andersson G, Carlbring P, Holmstrom A, Sparthan E, Furmark T, et al (2006) Internet-based self-help with therapist feedback and in vivo group exposure for social phobia: a randomized controlled trial. J Consult Clin Psychol 74: 677–686. 16881775 10.1037/0022-006X.74.4.677

[50] Berger T, Hohl E, Caspar F (2009) Internet-based treatment for social phobia: a randomized controlled trial. J Clin Psychol 65: 1021–1035. 19437505 10.1002/jclp.20603

[51] Titov N, Andrews G, Schwencke G, Drobny J, Einstein D (2008) Shyness 1: distance treatment of social phobia over the internet. Aust N Z J Psychiatry 42: 585–594. 18612862 10.1080/00048670802119762

[52] Titov N, Andrews G, Schwencke G (2008) Shyness 2: treating social phobia online: replication and extension. Aust N Z J Psychiatry 42: 595–605. 18612863 10.1080/00048670802119820

[53] Titov N, Andrews G, Choi I, Schwencke G, Mahoney A (2008) Shyness 3: randomized controlled trial of guided versus unguided internet-based CBT for social phobia. Aust N Z J Psychiatry 42: 1030–1040. 19016091 10.1080/00048670802512107

[54] Andersson G, Carlbring P, Furmark T (2012) S. O. F. I. E. Research Group (2012) Therapist experience and knowledge acquisition in internet-delivered CBT for social anxiety disorder: a randomized controlled trial. PLoS One 7: e37411. 22649526 10.1371/journal.pone.0037411 PMC3359350

[55] Carlbring P, Gunnarsdottir M, Hedensjo L, Andersson G, Ekselius L, et al (2007) Treatment of social phobia: randomised trial of internet-delivered cognitive-behavioural therapy with telephone support. Br J Psychiatry 190: 123–128. 17267928 10.1192/bjp.bp.105.020107

[56] Furmark T, Carlbring P, Hedman E, Sonnenstein A, Clevberger P, et al (2009) Guided and unguided self-help for social anxiety disorder: randomised controlled trial. Br J Psychiatry 195: 440–447. 19880935 10.1192/bjp.bp.108.060996

[57] Neubauer K, von Auer M, Murray E, Petermann F, Helbig-Lang S, et al (2013) Internet-delivered attention modification training as a treatment for social phobia: a randomized controlled trial. Behav Res Ther 51: 87–97. 23262116 10.1016/j.brat.2012.10.006

[58] Carlbring P, Nordgren LB, Furmark T, Andersson G (2009) Long-term outcome of Internet-delivered cognitive-behavioural therapy for social phobia: a 30-month follow-up. Behav Res Ther 47: 848–850. 19631312 10.1016/j.brat.2009.06.012

[59] Hedman E, Furmark T, Carlbring P, Ljotsson B, Ruck C, et al (2011) A 5-year follow-up of internet-based cognitive behavior therapy for social anxiety disorder. J Med Internet Res 13: e39. 21676694 10.2196/jmir.1776 PMC3221374

[60] Bergstrom J, Andersson G, Ljotsson B, Ruck C, Andreewitch S, et al (2010) Internet-versus group-administered cognitive behaviour therapy for panic disorder in a psychiatric setting: a randomised trial. BMC Psychiatry 10: 54. 20598127 10.1186/1471-244X-10-54 PMC2910662

[61] Wims E, Titov N, Andrews G, Choi I (2010) Clinician-assisted internet-based treatment is effective for panic: a randomized controlled trial. Aust N Z J Psychiatry 44: 599–607. 20560847 10.3109/00048671003614171

[62] Carlbring P, Bohman S, Brunt S, Buhrman M, Westling BE, et al (2006) Remote treatment of panic disorder: a randomized trial of internet-based cognitive behavior therapy supplemented with telephone calls. Am J Psychiatry 163: 2119–2125. 17151163 10.1176/ajp.2006.163.12.2119

[63] Carlbring P, Nilsson-Ihrfelt E, Waara J, Kollenstam C, Buhrman M, et al (2005) Treatment of panic disorder: live therapy vs. self-help via the Internet. Behav Res Ther 43: 1321–1333. 16086983 10.1016/j.brat.2004.10.002

[64] Klein B, Richards JC, Austin DW (2006) Efficacy of internet therapy for panic disorder. J Behav Ther Exp Psychiatry 37: 213–238. 16126161 10.1016/j.jbtep.2005.07.001

[65] Andersson G, Paxling B, Roch-Norlund P, Ostman G, Norgren A, et al (2012) Internet-based psychodynamic versus cognitive behavioral guided self-help for generalized anxiety disorder: a randomized controlled trial. Psychother Psychosom 81: 344–355. 22964540 10.1159/000339371

[66] Paxling B, Almlov J, Dahlin M, Carlbring P, Breitholtz E, et al (2011) Guided internet-delivered cognitive behavior therapy for generalized anxiety disorder: a randomized controlled trial. Cogn Behav Ther 40: 159–173. 21770848 10.1080/16506073.2011.576699

[67] Robinson E, Titov N, Andrews G, McIntyre K, Schwencke G, et al (2010) Internet treatment for generalized anxiety disorder: a randomized controlled trial comparing clinician vs. technician assistance. PLoS One 5: e10942. 20532167 10.1371/journal.pone.0010942 PMC2880592

[68] Titov N, Andrews G, Robinson E, Schwencke G, Johnston L, et al (2009) Clinician-assisted internet-based treatment is effective for generalized anxiety disorder: randomized controlled trial. Aust N Z J Psychiatry 43: 905–912. 10.1080/00048670902873722 19440890

[69] Andersson G, Waara J, Jonsson U, Malmaeus F, Carlbring P, et al (2009) Internet-based self-help versus one-session exposure in the treatment of spider phobia: a randomized controlled trial. Cogn Behav Ther 38: 114–120. 20183690 10.1080/16506070902931326

[70] Spence J, Titov N, Dear BF, Johnston L, Solley K, et al (2011) Randomized controlled trial of internet-delivered cognitive behavioral therapy for posttraumatic stress disorder. Depress Anxiety 28: 541–550. 21721073 10.1002/da.20835

[71] Andersson E, Enander J, Andren P, Hedman E, Ljotsson B, et al (2012) Internet-based cognitive behaviour therapy for obsessive-compulsive disorder: a randomized controlled trial. Psychol Med 42: 2193–2203. 22348650 10.1017/S0033291712000244 PMC3435873

[72] Johnston L, Titov N, Andrews G, Spence J, Dear BF (2011) A RCT of a transdiagnostic internet-delivered treatment for three anxiety disorders: examination of support roles and disorder-specific outcomes. PLoS One 6: e28079. 22132216 10.1371/journal.pone.0028079 PMC3223218

[73] Titov N, Andrews G, Johnston L, Robinson E, Spence J (2010) Transdiagnostic internet treatment for anxiety disorders: a randomized controlled trial. Behav Res Ther 48: 890–899. 20561606 10.1016/j.brat.2010.05.014

[74] Carlbring P, Maurin L, Torngren C, Linna E, Eriksson T, et al (2011) Individually-tailored, Internet-based treatment for anxiety disorders: a randomized controlled trial. Behav Res Ther 49: 18–24. 21047620 10.1016/j.brat.2010.10.002

[75] Bell CJ, Colhoun HC, Carter FA, Frampton CM (2012) Effectiveness of computerised cognitive behaviour therapy for anxiety disorders in secondary care. Aust N Z J Psychiatry 46: 630–640. 22327097 10.1177/0004867412437345

[76] Titov N, Dear BF, Schwencke G, Andrews G, Johnston L, et al (2011) Transdiagnostic internet treatment for anxiety and depression: a randomised controlled trial. Behav Res Ther 49: 441–452. 21679925 10.1016/j.brat.2011.03.007

[77] Newby JM, Mackenzie A, Williams AD, McIntyre K, Watts S, et al. (2013) Internet cognitive behavioural therapy for mixed anxiety and depression: a randomized controlled trial and evidence of effectiveness in primary care. Psychol Med: 1–14. 10.1017/S0033291713000111 23419552

[78] March S, Spence SH, Donovan CL (2009) The efficacy of an internet-based cognitive-behavioral therapy intervention for child anxiety disorders. J Pediatr Psychol 34: 474–487. 18794187 10.1093/jpepsy/jsn099

[79] Hedman E, Andersson E, Ljotsson B, Andersson G, Ruck C, et al (2011) Cost-effectiveness of Internet-based cognitive behavior therapy vs. cognitive behavioral group therapy for social anxiety disorder: results from a randomized controlled trial. Behav Res Ther 49: 729–736. 21851929 10.1016/j.brat.2011.07.009

[80] Hollinghurst S, Peters TJ, Kaur S, Wiles N, Lewis G, et al (2010) Cost-effectiveness of therapist-delivered online cognitive-behavioural therapy for depression: randomised controlled trial. Br J Psychiatry 197: 297–304. 20884953 10.1192/bjp.bp.109.073080

[81] Andersson G, Carlbring P, Berger T, Almlöv J, Cuijpers P (2009) What makes Internet therapy work? Cogn Behav Ther 38 Suppl 155–60. 19675956 10.1080/16506070902916400

[82] Kimmelman J, Weijer C, Meslin EM (2009) Helsinki discords: FDA, ethics, and international drug trials. Lancet 373: 13–14. 19121708 10.1016/S0140-6736(08)61936-4

[83] Hey SP, Weijer C (2013) Assay sensitivity and the epistemic contexts of clinical trials. Perspect Biol Med 56: 1–17. 23748523 10.1353/pbm.2013.0002

[84] Rothmann MD, Wiens BL, Chan ISF (2011) Design and analysis of non-inferiority trials. Boca Raton, FL: CRC Press. 438 p.

[85] National Institute for Health and Care Excellence (2009) Depression: the treatment and management of depression in adults (partial update of NICE clinical guideline 23). Clinical guideline 90 . Available from: http://guidance.nice.org.uk/cg90.

[86] National Institute for Health and Care Excellence (2013) Social anxiety disorder: recognition, assessment and treatment. Clinical guideline 159 . Available: http://guidance.nice.org.uk/cg159. 31869048

[87] Hedman E, Ljótsson B, Lindefors N (2012) Cognitive behavior therapy via the Internet: a systematic review of applications, clinical efficacy and cost–effectiveness. Expert Rev Pharmacoecon Outcomes Res 12: 745–764. 23252357 10.1586/erp.12.67

[88] Chambless DL, Baker MJ, Baucom DH, Beutler LE, Calhoun KS, et al (1998) Update on empirically validated therapies, II. Clinical Psychologist 51: 3–16.

[89] Sloan DM, Gallagher MW, Feinstein BA, Lee DJ, Pruneau GM (2011) Efficacy of telehealth treatments for posttraumatic stress-related symptoms: a meta-analysis. Cogn Behav Ther 40: 111–125. 21547778 10.1080/16506073.2010.550058

[90] Arnberg FK, Alaie I, Parling T, Jonsson U (2013) Recent randomized controlled trials of psychological interventions in healthcare: a review of their quantity, scope, and characteristics. J Psychosom Res 75: 401–408. 24182626 10.1016/j.jpsychores.2013.08.019

[91] Barak A, Hen L, Boniel-Nissim M, Shapira N (2008) A comprehensive review and a meta-analysis of the effectiveness of Internet-based psychotherapeutic interventions. J Technol Hum Serv 26: 109–160.

[92] Carlbring P, Westling BE, Ljungstrand P, Ekselius L, Andersson G (2001) Treatment of panic disorder via the Internet: a randomized trial of a self-help program. Behav Ther 32: 751–764.

[93] Peterson AL, Roache JD, Raj J, Young-McCaughan S, for the STRONG STAR Consortium The need for expanded monitoring of adverse events in behavioral health clinical trials. Contemp Clin Trials 34: 152–154. 23117077 10.1016/j.cct.2012.10.009

[94] Jonsson U, Alaie I, Parling T, Arnberg FK (2014) Reporting of harms in randomized controlled trials of psychological interventions for mental and behavioral disorders: A review of current practice. Contemp Clin Trials 38: 1–8. 24607768 10.1016/j.cct.2014.02.005

[95] Palmqvist B, Carlbring P, Andersson G (2007) Internet-delivered treatments with or without therapist input: does the therapist factor have implications for efficacy and cost? Expert Rev Pharmacoecon Outcomes Res 7: 291–297. 20528315 10.1586/14737167.7.3.291

[96] Johansson R, Andersson G (2012) Internet-based psychological treatments for depression. Expert Rev Neurother 12: 861–870. 22853793 10.1586/ern.12.63

[97] Boettcher J, Leek L, Matson L, Holmes EA, Browning M, et al (2013) Internet-based attention bias modification for social anxiety: a randomised controlled comparison of training towards negative and training towards positive cues. PLoS One 8: e71760. 24098630 10.1371/journal.pone.0071760 PMC3787061

[98] Hakamata Y, Lissek S, Bar-Haim Y, Britton JC, Fox NA, et al (2010) Attention bias modification treatment: a meta-analysis toward the establishment of novel treatment for anxiety. Biol Psychiatry 68: 982–990. 20887977 10.1016/j.biopsych.2010.07.021 PMC3296778

[99] Osenbach JE, O'Brien KM, Mishkind M, Smolenski DJ (2013) Synchronous telehealth technologies in psychotherapy for depression: a meta-analysis. Depress Anxiety 30: 1058–1067. 23922191 10.1002/da.22165

[100] Greenland S, O'Rourke K (2001) On the bias produced by quality scores in meta-analysis, and a hierarchical view of proposed solutions. Biostatistics 2: 463–471. 12933636 10.1093/biostatistics/2.4.463

