# Supplementary material for: Internet-Delivered Psychological Treatments for Mood and Anxiety Disorders: A Systematic Review of Their Efficacy, Safety, and Cost-Effectiveness
Source: PLoS One. 2014 May 20;9(5):e98118. doi: 10.1371/journal.pone.0098118 (PMC4028301; doi:10.1371/journal.pone.0098118)
Supplement: Appendix S1 — Detailed search strategy. (DOCX) [file pone.0098118.s001.docx]

**Table of Contents**

**Database search Page**

Cinahl via EBSCO 4 March 2013 1

Cochrane Library via Wiley 4 March 2013 (CDSR, DARE & HTA) 2

Psychology and behavioral sciences collection via EBSCO 5 March 2013 3

PsycInfo via EBSCO 5 March 2013 4

Pubmed via NLM 4 March 2013 5

| Cinahl via EBSCO 4 March 2013 | | |
| --- | --- | --- |
|  | **Search terms** | **Items found** |
| Population: People with mood and anxiety disorders | | |
|  | (MH "Affective Disorders") OR (MH "Depression") OR (MH "Premenstrual Dysphoric Disorder") OR (MH "Seasonal Affective Disorder") OR (MH "Depression, Postpartum") OR (MH "Depression, Reactive") OR (MH "Dysthymic Disorder") OR (MH "Anxiety Disorders") OR (MH "Generalized Anxiety Disorder") OR (MH "Separation Anxiety") OR (MH "Obsessive-Compulsive Disorder") OR (MH "Body Dysmorphic Disorder") OR (MH "Panic Disorder") OR (MH "Phobic Disorders") OR (MH "Acrophobia") OR (MH "Agoraphobia") OR (MH "Claustrophobia") OR (MH "Dental Anxiety") OR (MH "Homophobia") OR (MH "Social Anxiety Disorders") OR (MH "Stress Disorders, Post-Traumatic") OR (MH "Stockholm Syndrome") OR (MH "Mental Disorders") OR (MH "Panic Disorder") OR (MH "Bipolar Disorder") OR (MH "Affective Disorders, Psychotic") OR (MH "Cyclothymic Disorder") OR (MH "Obsessive-Compulsive Disorder") OR TI (Anxiety OR Depression OR Depressive OR Depressed OR Panic OR Phobic* OR phobia OR "Obsessive compulsive" OR "Mood disorder*" OR "compulsive hoarding" OR "post-traumatic stress" OR "anxiety disorder*" OR bipolar OR dysthymia OR Cyclothymic) | 83030 |
| Intervention: Internet | | |
|  | (MH "Online Services") OR (MH "Electronic Mail") OR (MH "Instant Messaging") OR (MH "Interactive Voice Response Systems") OR (MH "Electronic Bulletin Boards") OR (MH "Internet") OR (MH "Computer Assisted Instruction") OR TI (Computer* OR Web OR Website* OR Internet OR Multimedia OR Interactive OR Computerized OR Computerised OR Online OR Smartphone OR Chatt OR "mobile phone" OR "e-mail" OR "Smartphone app*" OR "Application for a smartphone" OR "Mobile phone application*" OR "cell phone application" OR "technology-assisted") | 56481 |
| Intervention: Psychological treatment | | |
|  | (MH "Psychotherapy") OR (MH "Psychotherapy, Brief") OR (MH "Psychotherapy, Group") OR (MH "Cognitive Therapy") OR (MH "Art Therapy") OR (MH "Behavior Modification") OR (MH "Crisis Intervention") OR (MH "Assertiveness Training") OR (MH "Behavior Contracting") OR (MH "Support Groups") OR (MH "Behavior Therapy") OR (MH "Relaxation Techniques") OR (MH "Meditation") OR (MH "Motivational Interviewing") OR MH "Early Intervention" OR MH "Psychologists" OR MH "Counseling" OR TI ("Cognitive behavior*" OR "Cognitive behaviour*" OR "dialectical behavior" OR "dialectical behaviour" OR "Acceptance and Commitment therapy" OR Ccbt OR CBT OR DBT OR ACT OR "minimal therapist support" OR "guided self-help" OR "supervised self-help" OR "minimal contact therap*" OR "interpersonal therapy" OR mindfulness OR relaxation OR psychodynamic OR "social rhythm therapy" OR therapy OR "motivational interviewing") | 108215 |
| Combined sets | | |
|  | 1 AND 2 AND 3 | 258 |
|  | 4 AND limiters: Limiters - Language: Danish, English, Norwegian, Swedish | 257 |
|  | 5 AND Limiters - Published Date from: 20120901-; | 10 |
| Study types | | |
|  | 6 AND ((MH "Systematic Review") OR (ZT "systematic review") OR (TI "systematic review") OR (TI "meta-analysis)) | 1 |
|  | 6 AND (MH "Randomized Controlled Trials" OR MH "Random Assignment" OR TI ("randomly assigned" OR randomiz* OR randomis* OR RCT)) | 2 |
|  | 6 NOT (7 OR 8) | 7 |

*Note.* The search result, usually found at the end of the documentation, forms the list of abstracts.

AB = Abstract. AU = Author. DE = Term from the thesaurus. MH = Term from the “Cinahl Headings” thesaurus. MM = Major Concept. TI = Title. TX = All Text. Performs a keyword search of all the  database's searchable fields. ZC = Methodology Index. * = Truncation. “ “ = Citation Marks; searches for an exact phrase.

| Cochrane Library via Wiley 4 March 2013 (CDSR, DARE & HTA) | | |
| --- | --- | --- |
|  | **Search terms** | **Items found** |
| Population: People with mood and anxiety disorders | | |
|  | "Anxiety Disorders"[Mesh] OR Mood Disorders[MeSH] | 11695 |
| Intervention: Internet | | |
|  | Therapy, Computer-Assisted[MeSH:NoExp] OR "Computer-Assisted Instruction"[Mesh] OR "Internet"[Mesh:NoExp] OR "Multimedia"[Mesh] OR "Electronic Mail"[MeSH] OR "Cellular Phone"[Mesh] | 2620 |
| Combined sets | | |
|  | 1 AND 2 | 189 |
|  | 3 AND limits 2012 | CDSR/0  DARE/0  CENTRAL/2  HTA/0 |

*Note.* The search result, usually found at the end of the documentation, forms the list of abstracts

[MeSH] = Term from the Medline controlled vocabulary, including terms found below this term in the MeSH hierarchy. [MeSH:NoExp] = Does not include terms found below this term in the MeSH hierarchy. “ “ = Citation Marks; searches for an exact phrase.

CDSR = Cochrane Database of Systematic Review. CENTRAL = Cochrane Central Register of Controlled Trials, “trials”. CRM = Method Studies. DARE = Database Abstracts of Reviews of Effects, “other reviews”. EED = Economic Evaluations. HTA = Health Technology Assessments

| Psychology and behavioral sciences collection via EBSCO 5 March 2013 | | |
| --- | --- | --- |
|  | **Search terms** | **Items found** |
| Population: People with mood and anxiety disorders | | |
|  | DE "DEPRESSION in adolescence" OR DE "DEPRESSION in children" OR DE "DEPRESSION in men" OR DE "DEPRESSION in old age" OR DE "DEPRESSION in women" OR DE "MENTAL depression" OR DE "AFFECTIVE disorders" OR DE "DYSTHYMIC disorder" OR DE "ANXIETY" OR DE "MANIC-depressive illness" OR DE "SEASONAL affective disorder" OR DE "ANXIETY" OR DE "FEAR of dentists" OR DE "FEAR of needles" OR DE "PANIC disorders" OR DE "PERFORMANCE anxiety" OR DE "SEPARATION anxiety" OR DE "SOCIAL anxiety" OR DE "SPEECH anxiety" OR DE "TEST anxiety" OR DE "PANIC disorders" OR DE "PANIC disorders" OR DE "AGORAPHOBIA" OR DE "GENERALIZED anxiety disorder" OR DE "POST-traumatic stress disorder" OR DE "POST-traumatic stress disorder in adolescence" OR DE "POST-traumatic stress disorder in children" OR DE "POST-traumatic stress disorder in old age" OR DE "BATTERED woman syndrome" OR DE "POST-traumatic stress disorder in children" OR DE "RAPE trauma syndrome" OR DE "MANIC-depressive illness" OR DE "PHOBIAS" OR DE "MENTAL illness " OR TI (Anxiety OR Depression OR Depressive OR Depressed OR Panic OR Phobic* OR phobia OR "Obsessive compulsive" OR "Mood disorder*" OR "compulsive hoarding" OR "post-traumatic stress" OR "anxiety disorder*" OR bipolar OR dysthymia OR cyclothymia) | 35,324 |
| Intervention: Internet | | |
|  | DE "INTERNET in medicine" OR DE "INTERNET in psychotherapy" OR DE "INTERNET" OR DE "WEB-based instruction" OR DE "E-mail" OR DE "COMPUTER-aided transcription systems" OR TI (Computer* OR Web OR Website* OR Internet OR Multimedia OR Interactive OR Computerized OR Computerised OR Online OR Smartphone OR Chatt OR "mobile phone" OR "e-mail" OR "Smartphone app*" OR "Application for a smartphone" OR "Mobile phone application*" OR "cell phone application" OR "technology-assisted") | 9,067 |
| Intervention: Psychological treatment | | |
|  | DE "COGNITIVE therapy" OR DE "PSYCHOTHERAPY" OR DE "ACCEPTANCE & commitment therapy" OR DE "COGNITIVE-analytic therapy" OR DE "COGNITIVE-experiential psychotherapy" OR DE "EXPOSURE therapy" OR DE "RATIONAL emotive behavior therapy" OR DE "SCHEMA-focused cognitive therapy" OR DE "ADOLESCENT psychotherapy" OR DE "ADVENTURE therapy" OR DE "ANGER management therapy" OR DE "ART therapy" OR DE "ASSERTIVENESS training" OR DE "BEHAVIOR therapy" OR DE "BRIEF psychotherapy" OR DE "CHILD psychotherapy" OR DE "CLIENT-centered psychotherapy" OR DE "COGNITIVE therapy" OR DE "COMMUNICATIVE psychotherapy" OR DE "CONJOINT therapy" OR DE "DESENSITIZATION (Psychotherapy)" OR DE "DEVELOPMENTAL therapy" OR DE "EMOTION-focused therapy" OR DE "EMOTIONAL Freedom Techniques" OR DE "ENVIRONMENTAL psychology" OR DE "ETHICAL therapy" OR DE "EXISTENTIAL psychotherapy" OR DE "FEELING therapy" OR DE "FEMINIST therapy" OR DE "FREE association (Psychology)" OR DE "GESTALT therapy" OR DE "GRIEF therapy" OR DE "GROUP psychotherapy" OR DE "HUMANISTIC psychotherapy" OR DE "IMPASSE (Psychotherapy)" OR DE "INSIGHT in psychotherapy" OR DE "INTERNET in psychotherapy" OR DE "INTERPERSONAL & social rhythm therapy" OR DE "INTERPERSONAL psychotherapy" OR DE "MOTION pictures in psychotherapy" OR DE "MULTIMODAL psychotherapy" OR DE "MULTIPLE psychotherapy" OR DE "NAIKAN psychotherapy" OR DE "PERSONAL construct therapy" OR DE "PROBLEM-solving therapy" OR DE "PSYCHIATRY -- Differential therapeutics" OR DE "PSYCHODYNAMIC psychotherapy" OR DE "SOLUTION-focused therapy" OR DE "STRATEGIC therapy" OR DE "SUPPORTIVE psychotherapy" OR DE "TELEPHONE in psychotherapy" OR DE "COUNSELING" OR DE "MENTAL health counseling" OR DE "PSYCHIATRIC treatment" OR DE "PSYCHOANALYSIS" OR DE "MENTAL health services" OR DE "RELAXATION" OR DE "MEDITATION" OR TI ("Cognitive behavior*" OR "Cognitive behaviour*" OR "dialectical behavior" OR "dialectical behaviour" OR "Acceptance and Commitment therapy" OR Ccbt OR CBT OR DBT OR ACT OR "minimal therapist support" OR "guided self-help" OR "supervised self-help" OR "minimal contact therap*" OR "interpersonal therapy" OR mindfulness OR relaxation OR psychodynamic OR "social rhythm therapy" OR therapy OR "motivational interviewing") | 36,909 |
| Combined sets | | |
|  | 1 AND 2 AND 3 | 457 |
|  | 4 AND Limiters - Published Date from: 20120901- | 25 |

*Note.* The search result, usually found at the end of the documentation, forms the list of abstracts.

AB = Abstract. AU = Author. DE = Term from the thesaurus. MH = Term from the “Cinahl Headings” thesaurus. MM = Major Concept. TI = Title. TX = All Text. Performs a keyword search of all the database's searchable fields. ZC = Methodology Index. * = Truncation. “ “ = Citation Marks; searches for an exact phrase.

| PsycInfo via EBSCO 5 March 2013 | | |
| --- | --- | --- |
|  | **Search terms** | **Items found** |
| Population: People with mood and anxiety disorders | | |
|  | DE "Anxiety" OR DE "Social Anxiety" OR DE "Anxiety Disorders" OR DE "Generalized Anxiety Disorder" OR DE "Panic" OR DE "Panic Attack" OR DE "Panic Disorder" OR DE "Phobias" OR DE "Acute Stress Disorder" OR DE "Castration Anxiety" OR DE "Death Anxiety" OR DE "Generalized Anxiety Disorder" OR DE "Obsessive Compulsive Disorder" OR DE "Panic Disorder" OR DE "Phobias" OR DE "Posttraumatic Stress Disorder" OR DE "Separation Anxiety" OR DE "Acrophobia" OR DE "Agoraphobia" OR DE "Claustrophobia" OR DE "Ophidiophobia" OR DE "School Phobia" OR DE "Social Phobia" OR DE "Affective Disorders" OR DE "Bipolar Disorder" OR DE "Major Depression" OR DE "Mania" OR DE "Dysthymic Disorder" OR DE "Anaclitic Depression" OR DE "Cyclothymic Personality" OR DE "Endogenous Depression" OR DE "Postpartum Depression" OR DE "Reactive Depression" OR DE "Treatment Resistant Depression" OR DE "Atypical Depression" OR DE "Depression (Emotion)" OR DE "Recurrent Depression" OR DE "Mental Health" OR TI (Anxiety OR Depression OR Depressive OR Depressed OR Panic OR Phobic* OR phobia OR "Obsessive compulsive" OR "Mood disorder*" OR "compulsive hoarding" OR "post-traumatic stress" OR "anxiety disorder*" OR bipolar OR dysthymia OR Cyclothymi*) | 245,678 |
| Intervention: Internet | | |
|  | DE "Online Therapy" OR DE "Internet" OR DE "Cellular Phones" OR DE "Computer Applications" OR DE "Computer Assisted Therapy" OR DE "Computer Assisted Instruction" OR DE "Online Social Networks" OR DE "Websites" OR TI (Computer* OR Web OR Website* OR Internet OR Multimedia OR Interactive OR Computerized OR Computerised OR Online OR Smartphone OR Chatt OR "mobile phone" OR "e-mail" OR "Smartphone app*" OR "Application for a smartphone" OR "Mobile phone application*" OR "cell phone application" OR "technology-assisted") | 59,753 |
| Intervention: Psychological treatment | | |
|  | DE "Online Therapy" OR DE "Psychotherapy" OR DE "Adlerian Psychotherapy" OR DE "Adolescent Psychotherapy" OR DE "Analytical Psychotherapy" OR DE "Autogenic Training" OR DE "Behavior Therapy" OR DE "Brief Psychotherapy" OR DE "Child Psychotherapy" OR DE "Client Centered Therapy" OR DE "Cognitive Behavior Therapy" OR DE "Conversion Therapy" OR DE "Eclectic Psychotherapy" OR DE "Emotion Focused Therapy" OR DE "Existential Therapy" OR DE "Experiential Psychotherapy" OR DE "Expressive Psychotherapy" OR DE "Eye Movement Desensitization Therapy" OR DE "Feminist Therapy" OR DE "Geriatric Psychotherapy" OR DE "Gestalt Therapy" OR DE "Group Psychotherapy" OR DE "Guided Imagery" OR DE "Hypnotherapy" OR DE "Individual Psychotherapy" OR DE "Insight Therapy" OR DE "Integrative Psychotherapy" OR DE "Interpersonal Psychotherapy" OR DE "Logotherapy" OR DE "Narrative Therapy" OR DE "Persuasion Therapy" OR DE "Primal Therapy" OR DE "Psychoanalysis" OR DE "Psychodrama" OR DE "Psychodynamic Psychotherapy" OR DE "Psychotherapeutic Counseling" OR DE "Rational Emotive Behavior Therapy" OR DE "Reality Therapy" OR DE "Relationship Therapy" OR DE "Solution Focused Therapy" OR DE "Supportive Psychotherapy" OR DE "Transactional Analysis" OR DE "Cognitive Therapy" OR DE "Conversion Therapy" OR DE "Dialectical Behavior Therapy" OR DE "Exposure Therapy" OR DE "Implosive Therapy" OR DE "Reciprocal Inhibition Therapy" OR DE "Systematic Desensitization Therapy" OR DE "Anger Control" OR DE "Acceptance and Commitment Therapy" OR DE "Cognitive Therapy" OR DE "Behavior Modification" OR DE "Counseling" OR DE "Self Help Techniques" OR DE "Group Counseling" OR TI ("Cognitive behavior*" OR "Cognitive behaviour*" OR "dialectical behavior" OR "dialectical behaviour" OR "Acceptance and Commitment therapy" OR Ccbt OR CBT OR DBT OR ACT OR "minimal therapist support" OR "guided self-help" OR "supervised self-help" OR "minimal contact therap*" OR "interpersonal therapy" OR mindfulness OR relaxation OR psychodynamic OR "social rhythm therapy" OR therapy OR "motivational interviewing") | 226,612 |
| Combined sets | | |
|  | 1 AND 2 AND 3 | 580 |
|  | 4 AND Limiters - Publication Year from: 2012-; Language: Danish, English, Norwegian, Swedish | 73 |
| Study types: Systematic reviews, RCTs | | |
|  | 5 AND ((ZC "systematic review") OR (TI "systematic review") OR (TI "meta-analysis")) | 3 |
|  | 5 AND (((ZC "treatment outcome/clinical trial" OR DE "Clinical Trials") AND TX "random*") OR TI ("randomly assigned" OR randomiz* OR randomis* OR RCT)) | 27 |
|  | 5 NOT (6 OR 7) | 43 |

*Note.*The search result, usually found at the end of the documentation, forms the list of abstracts.

AB = Abstract. AU = Author. DE = Term from the thesaurus. MH = Term from the “Cinahl Headings” thesaurus. MM = Major Concept. TI = Title. TX = All Text. Performs a keyword search of all the  database's searchable fields. ZC = Methodology Index. * = Truncation. “ “ = Citation Marks; searches for an exact phrase.

| Pubmed via NLM 4 March 2013 | | |
| --- | --- | --- |
|  | **Search terms** | **Items found** |
| Population: People with mood and anxiety disorders | | |
|  | Anxiety[MeSH] OR "Anxiety Disorders"[Mesh] OR "Depression"[Mesh] OR "Depressive Disorder"[Mesh] OR Mood Disorders[MeSH] OR "Cyclothymic Disorder"[Mesh] OR "Bipolar Disorder"[Mesh] OR Anxiety[tiab] OR Depression[tiab] OR Depressive[tiab] OR Depressed[tiab] OR Panic[tiab] OR Phobic*[tiab] OR phobia[tiab] OR "Obsessive compulsive"[tiab] OR "Mood disorder*"[tiab] OR "compulsive hoarding"[tiab] OR "post-traumatic stress"[tiab] OR "anxiety disorder*"[tiab] OR bipolar[tiab] OR dysthymia[tiab] OR cyclothymic[tiab] OR bipolar[tiab] | 454883 |
| Intervention: Internet | | |
|  | "Therapy, Computer-Assisted"[MeSH] OR "Computer-Assisted Instruction"[Mesh] OR "Internet"[Mesh:NoExp] OR "Multimedia"[Mesh] OR "Electronic Mail"[MeSH] OR "Cellular Phone"[Mesh] OR Computer[ti] OR Web[ti] OR Website[ti] OR websites[ti] OR Internet[ti] OR Multimedia[ti] OR Interactive[ti] OR Computerized[ti] OR Computerised[ti] OR Online[ti] OR Smartphone[ti] OR Chatt[ti] OR "mobile phone"[ti] OR e-mail[ti] OR "Smartphone app*"[ti] OR "Application for a smartphone"[ti] OR "Mobile phone application*"[ti] OR "cell phone application"[ti] OR "technology-assisted"[ti] OR ((Computer[tiab] OR Web[tiab] OR Website[tiab] OR websites[tiab] OR Internet[tiab] OR Multimedia[tiab] OR Interactive[tiab] OR Computerized[tiab] OR Computerised[tiab] OR Online[tiab] OR Smartphone[tiab] OR Chatt[tiab] OR "mobile phone"[tiab] OR e-mail[tiab] OR "Smartphone app*"[tiab] OR "Application for a smartphone"[tiab] OR "Mobile phone application*"[tiab] OR "cell phone application"[tiab] OR "technology-assisted"[tiab]) NOT (Medline[SB] OR oldmedline[SB])) | 178919 |
| Intervention: Psychological treatment | | |
|  | "Psychotherapy"[Mesh] OR "Behavior Therapy"[Mesh] OR Cognitive Therapy[MeSH] OR "Directive Counseling"[Mesh] OR "Remote Consultation"[Mesh] OR "Self Care"[Mesh:NoExp] OR "therapy"[Subheading:NoExp] OR "Motivation"[Mesh] OR "Relaxation Therapy"[Mesh] OR Cognitive behavior[tiab] OR "Cognitive behaviour"[tiab] OR Cognitive behavioral[tiab] OR "Cognitive behavioural"[tiab] OR "dialectical behavior"[tiab] OR "dialectical behaviour"[tiab] OR "Acceptance and Commitment therapy"[tiab] OR Ccbt[tiab] OR CBT[tiab] OR DBT[tiab] OR ACT[tiab] OR "minimal therapist support"[tiab] OR "guided self-help"[tiab] OR "supervised self-help"[tiab] OR "minimal contact therap*"[tiab] OR "interpersonal therapy"[tiab] OR mindfulness[tiab] OR relaxation[tiab] OR psychodynamic[tiab] OR "social rhythm therapy"[tiab] OR ((therapy[tiab] OR "motivational interviewing"[tiab] OR intervention[tiab] OR interventions[tiab] OR treatment[tiab]) NOT (Medline[SB] OR Oldmedline[SB])) | 2051186 |
| Combined sets | | |
|  | 1 AND 2 AND 3 | 1559 |
|  | 4 AND ("english"[Language] OR "swedish"[Language] OR "danish"[Language] OR "norwegian"[Language]) | 1487 |
|  | 5 AND Filters activated: Publication date from 2012/09/01 | 295 |
| Study types: systematic reviews, RCTs | | |
|  | 5 AND systematic[SB] | 34 |
|  | 5 AND ("Randomized Controlled Trial" [Publication Type] OR ((randomized[tiab] OR randomization[tiab] OR randomized[tiab] OR randomization[tiab] OR "randomly assigned"[tiab] OR controlled[tiab]) AND (trial[tiab] OR study[tiab] OR cohort[tiab])) OR (randomized[ti] OR randomized[ti] OR RCT[ti])) | 91 |
|  | 5 NOT (7 OR 8) | 178 |

*Note*. The search result, usually found at the end of the documentation, forms the list of abstracts.

[MeSH] = Term from the Medline controlled vocabulary, including terms found below this term in the MeSH hierarchy. [MeSH:NoExp] = Does not include terms found below this term in the MeSH hierarchy. [MAJR] = MeSH Major Topic. [TIAB] = Title or abstract. [TI] = Title. [AU] = Author. [TW] = Text Word. Systematic. [SB] = Filter for retrieving systematic reviews. * = Truncation. “ “ = Citation Marks; searches for an exact phrase.
