# Supplementary material for: Internet-Delivered Psychological Treatments for Mood and Anxiety Disorders: A Systematic Review of Their Efficacy, Safety, and Cost-Effectiveness
Source: PLoS One. 2014 May 20;9(5):e98118. doi: 10.1371/journal.pone.0098118 (PMC4028301; doi:10.1371/journal.pone.0098118)
Supplement: Appendix S2 — List of reports excluded after full-text reading. (DOCX) [file pone.0098118.s002.docx]

**Table of Contents**

**Reasons for exclusion Page**

Irrelevant efficacy trials, 173 reports 1

Population not diagnosed or otherwise not eligible, 46 reports 1

Secondary sources, 43 reports 2

Research question not relevant, 40 reports 4

Intervention not via Internet or otherwise not eligible, 35 reports 5

Research design, 7 reports 7

Protocol, 2 reports 7

Efficacy trials with high risk of bias, 13 reports 7

Mood disorders 7

Social phobia 7

Panic disorder 7

Posttraumatic stress disorder 7

Children and adolescents 7

Irrelevant cost-effectiveness studies, 13 reports 8

Cost-effectiveness studies with high risk of bias, 3 reports 8

# Irrelevant efficacy trials, 173 reports

## Population not diagnosed or otherwise not eligible, 46 reports

Andersson G, Bergstrom J, Hollandare F, et al. Internet-based self-help for depression: Randomised controlled trial. Br J Psychiatry 2005;187:456-461.

Christensen H, Griffiths KM, Jorm AF. Delivering interventions for depression by using the internet: Randomised controlled trial. BMJ 2004;328:265-268.

Clarke G, Eubanks D, Reid E, et al. Overcoming depression on the internet (odin) (2): A randomized trial of a self-help depression skills program with reminders. J Med Internet Res 2005;7:e16.

Clarke G, Kelleher C, Hornbrook M, et al. Randomized effectiveness trial of an internet, pure self-help, cognitive behavioral intervention for depressive symptoms in young adults. Cogn Behav Ther 2009;38:222-234.

Clarke G, Reid E, Eubanks D, et al. Overcoming depression on the internet (odin): A randomized controlled trial of an internet depression skills intervention program. J Med Internet Res 2002;4:E14.

de Graaf LE, Gerhards SA, Arntz A, et al. One-year follow-up results of unsupported online computerized cognitive behavioural therapy for depression in primary care: A randomized trial. J Behav Ther Exp Psychiatry 2011;42:89-95.

de Graaf LE, Gerhards SAH, Arntz A, et al. Clinical effectiveness on online computerised cognitive-behavioural therapy without support for depression in primary care: Randomised trial. Br J Psychiatry 2009;195:73-80.

Farrer L, Christensen H, Griffiths KM, Mackinnon A. Internet-based cbt for depression with and without telephone tracking in a national helpline: Randomised controlled trial. PLoS One 2011;6:e28099.

Garnefski N, Kraaij V, Schroevers M. Effects of a cognitive behavioral self-help program on depressed mood for people with acquired chronic physical impairments: A pilot randomized controlled trial. Patient Educ Couns 2011;85:304-307.

Grassi A, Gaggioli A, Riva G. New technologies to manage exam anxiety. Studies in health technology and informatics 2011;167:57-62.

Griffiths KM, Mackinnon AJ, Crisp DA, et al. The effectiveness of an online support group for members of the community with depression: A randomised controlled trial. PLoS One 2012;7:e53244.

Grime PR. Computerized cognitive behavioural therapy at work: A randomized controlled trial in employees with recent stress-related absenteeism. Occup Med (Lond) 2004;54:353-359.

Hedman E, Andersson G, Andersson E, et al. Internet-based cognitive-behavioural therapy for severe health anxiety: Randomised controlled trial. Br J Psychiatry 2011;198:230-236.

Hoek W, Schuurmans J, Koot HM, Cuijpers P. Effects of internet-based guided self-help problem-solving therapy for adolescents with depression and anxiety: A randomized controlled trial. PLoS One 2012;7:e43485.

Hollandare F, Johnsson S, Randestad M, et al. Randomized trial of internet-based relapse prevention for partially remitted depression. Acta Psychiatr Scand 2011;124:285-294.

Kauer SD, Reid SC, Crooke AH, et al. Self-monitoring using mobile phones in the early stages of adolescent depression: Randomized controlled trial. J Med Internet Res 2012;14:e67.

Kenardy J, McCafferty K, Rosa V. Internet-deliverered indicated prevention for anxiety disorders: A randomized controlled trial. Behav Cogn Psychother 2003;31:279-289.

Kenardy J, McCafferty K, Rosa V. Internet-delivered indicated prevention for anxiety disorders: Six-month follow-up. Clinical Psychologist 2006;10:39-42.

Kersting A, Kroker K, Schlicht S, et al. Efficacy of cognitive behavioral internet-based therapy in parents after the loss of a child during pregnancy: Pilot data from a randomized controlled trial. Arch Womens Ment Health 2011;14:465-477.

Knaevelsrud C, Maercker A. Internet-based treatment for ptsd reduces distress and facilitates the development of a strong therapeutic alliance: A randomized controlled clinical trial. BMC Psychiatry 2007;7:13.

Knaevelsrud C, Maercker A. Long-term effects of an internet-based treatment for posttraumatic stress. Cogn Behav Ther 2010;39:72-77.

Kraaij V, van Emmerik A, Garnefski N, et al. Effects of a cognitive behavioral self-help program and a computerized structured writing intervention on depressed mood for hiv-infected people: A pilot randomized controlled trial. Patient Educ Couns 2010;80:200-204.

Kroenke K, Theobald D, Wu J, et al. Effect of telecare management on pain and depression in patients with cancer: A randomized trial. JAMA 2010;304:163-171.

Lange A, Rietdijk D, Hudcovicova M, et al. Interapy: A controlled randomized trial of the standardized treatment of posttraumatic stress through the internet. J Consult Clin Psychol 2003;71:901-909.

Lange A, van de Ven JP, Schrieken B, Emmelkamp PM. Interapy, treatment of posttraumatic stress through the internet: A controlled trial. J Behav Ther Exp Psychiatry 2001;32:73-90.

Lintvedt OK, Griffiths KM, Sorensen K, et al. Evaluating the effectiveness and efficacy of unguided internet-based self-help intervention for the prevention of depression: A randomized controlled trial. Clin Psychol Psychother 2011.

Mackinnon A, Griffiths KM, Christensen H. Comparative randomised trial of online cognitive-behavioural therapy and an information website for depression: 12-month outcomes. Br J Psychiatry 2008;192:130-134.

Meyer B, Berger T, Caspar F, et al. Effectiveness of a novel integrative online treatment for depression (deprexis): Randomized controlled trial. J Med Internet Res 2009;11:e15.

Morgan AJ, Jorm AF, Mackinnon AJ. Email-based promotion of self-help for subthreshold depression: Mood memos randomised controlled trial. Br J Psychiatry 2012;200:412-418.

Moritz S, Schilling L, Hauschildt M, et al. A randomized controlled trial of internet-based therapy in depression. Behav Res Ther 2012;50:513-521.

O'Kearney R, Gibson M, Christensen H, Griffiths KM. Effects of a cognitive-behavioural internet program on depression, vulnerability to depression and stigma in adolescent males: A school-based controlled trial. Cogn Behav Ther 2006;35:43-54.

O'Kearney R, Kang K, Christensen H, Griffiths K. A controlled trial of a school-based internet program for reducing depressive symptoms in adolescent girls. Depress Anxiety 2009;26:65-72.

O'Kearney R, Kang K, Gibson M, et al. A cbt internet program for depression in adolescents (moodgym): Effects on depressive symptoms, attributional style, self-esteem and beliefs about depression. In: Einstein DA, editor. Innovations and advances in cognitive behaviour therapy. Bowen Hills, QLD,Australia: Australian Academic Press; 2007. p. 197-204.

Orbach G, Lindsay S, Grey S. A randomised placebo-controlled trial of a self-help internet-based intervention for test anxiety. Behav Res Ther 2007;45:483-496.

Rosmarin DH, Pargament KI, Pirutinsky S, Mahoney A. A randomized controlled evaluation of a spiritually integrated treatment for subclinical anxiety in the jewish community, delivered via the internet. J Anxiety Disord 2010;24:799-808.

Ruwaard J, Broeksteeg J, Schrieken B, et al. Web-based therapist-assisted cognitive behavioral treatment of panic symptoms: A randomized controlled trial with a three-year follow-up. J Anxiety Disord 2010;24:387-396.

Ruwaard J, Schrieken B, Schrijver M, et al. Standardized web-based cognitive behavioural therapy of mild to moderate depression: A randomized controlled trial with a long-term follow-up. Cogn Behav Ther 2009;38:206-221.

Sheeber LB, Seeley JR, Feil EG, et al. Development and pilot evaluation of an internet-facilitated cognitive-behavioral intervention for maternal depression. J Consult Clin Psychol 2012.

Silfvernagel K, Carlbring P, Kabo J, et al. Individually tailored internet-based treatment for young adults and adults with panic attacks: Randomized controlled trial. J Med Internet Res 2012;14:e65.

Spek V, Cuijpers P, Nyklicek I, et al. One-year follow-up results of a randomized controlled clinical trial on internet-based cognitive behavioural therapy for subthreshold depression in people over 50 years. Psychol Med 2008;38:635-639.

Spek V, Nyklíček I, Smits N, et al. Internet-based cognitive behavioural therapy for subthreshold depression in people over 50 years old: A random controlled clinical trial. Psychol Med 2007;37:1797-1806.

Steinmetz SE, Benight CC, Bishop SL, James LE. My disaster recovery: A pilot randomized controlled trial of an internet intervention. Anxiety Stress Coping 2011.

Thompson NJ, Walker ER, Obolensky N, et al. Distance delivery of mindfulness-based cognitive therapy for depression: Project uplift. Epilepsy & behavior : E&B 2010;19:247-254.

van Bastelaar KM, Pouwer F, Cuijpers P, et al. Web-based depression treatment for type 1 and type 2 diabetic patients: A randomized, controlled trial. Diabetes care 2011;34:320-325.

Wagner B, Knaevelsrud C, Maercker A. Internet-based cognitive-behavioral therapy for complicated grief: A randomized controlled trial. Death Stud 2006;30:429-453.

Warmerdam L, van Straten A, Twisk J, et al. Internet-based treatment for adults with depressive symptoms: Randomized controlled trial. J Med Internet Res 2008;10:e44.

## Secondary sources, 43 reports

Computer-assisted cognitive-behavioral treatment for depression. Lansdale: HAYES, Inc.; 2009.

Andersson G. Online cognitive behavioural therapy is effective for depression in primary care. Evidence Based Mental Health 2010;13:50.

Amstadter AB, Broman-Fulks J, Zinzow H, et al. Internet-based interventions for traumatic stress-related mental health problems: A review and suggestion for future research. Clin Psychol Rev 2009;29:410-420.

Andersson G, Cuijpers P. Internet-based and other computerized psychological treatments for adult depression: A meta-analysis. Cogn Behav Ther 2009;38:196-205.

Andrews G, Cuijpers P, Craske MG, et al. Computer therapy for the anxiety and depressive disorders is effective, acceptable and practical health care: A meta-analysis (structured abstract). PLoS One [serial on the Internet]. 2010; (10): Available from: http://www.mrw.interscience.wiley.com/cochrane/cldare/articles/DARE-12011000359/frame.html.

Barak A, Hen L, Boniel-Nissim M, Shapira N. A comprehensive review and a meta-analysis of the effectiveness of internet-based psychotherapeutic interventions. J Technol Hum Serv 2008;26:109-160.

Bee PE, Bower P, Lovell K, et al. Psychotherapy mediated by remote communication technologies: A meta-analytic review. BMC Psychiatry 2008;8:60.

Bergström J, Holländare F, Carlbring P, et al. Treatment of depression via the internet: A randomized trial of a self-help programme. J Telemed Telecare 2003;9:85.

Bickel KW. An empirical test of calm for pd: A computer-administered learning module for panic disorder. Dissertation Abstracts International: Section B: The Sciences and Engineering 2008;68:5558.

Bower P, Kontopantelis E, Sutton A, et al. Influence of initial severity of depression on effectiveness of low intensity interventions: Meta-analysis of individual patient data. BMJ 2013;346:f540.

Calear AL, Christensen H. Review of internet-based prevention and treatment programs for anxiety and depression in children and adolescents. Med J Aust 2010;192:S12-14.

Choi M, Kong S, Jung D. Computer and internet interventions for loneliness and depression in older adults: A meta-analysis. Healthc Inform Res 2012;18:191-198.

Christensen H, Petrie K. State of the e-mental health field in australia: Where are we now? Aust N Z J Psychiatry 2013;47:117-120.

Corrieri S, Heider D, Conrad I, et al. School-based prevention programs for depression and anxiety in adolescence: A systematic review. Health promotion international 2013.

Cuijpers P, Marks IM, van Straten A, et al. Computer-aided psychotherapy for anxiety disorders: A meta-analytic review (structured abstract). Cogn Behav Ther [serial on the Internet]. 2009; (2): Available from: http://www.mrw.interscience.wiley.com/cochrane/cldare/articles/DARE-12009108805/frame.html.

Cuijpers P, Schuurmans J. Self-help interventions for anxiety disorders: An overview. Curr Psychiatry Rep 2007;9:284-290.

Cuijpers P, van Straten A, Andersson G. Internet-administered cognitive behavior therapy for health problems: A systematic review (structured abstract). Journal of Behavioral Medicine [serial on the Internet]. 2008; (2): Available from: http://www.mrw.interscience.wiley.com/cochrane/cldare/articles/DARE-12008105089/frame.html.

Donovan CL, Newall C, Hudson JL. Online cognitive-behaviour therapy is similarly effective to clinic-based cbt for reducing adolescent anxiety. Evidence Based Mental Health 2012;15:49-49.

Ehrenreich B, Righter B, Rocke DA, et al. Are mobile phones and handheld computers being used to enhance delivery of psychiatric treatment? A systematic review. J Nerv Ment Dis 2011;199:886-891.

Ferriter M, Kaltenthaler E, Parry G, Beverley C. Computerised cbt: A review. Mental health today (Brighton, England) 2008:30-31.

Foroushani PS, Schneider J, Assareh N. Meta-review of the effectiveness of computerised cbt in treating depression. BMC Psychiatry 2011;11:131.

Garcia-Lizana F, Munoz-Mayorga I. Telemedicine for depression: A systematic review. Perspect Psychiatr Care 2010;46:119-126.

Griffiths KM. Sparx computerised cbt is as effective as usual care for mild-to-moderate depression in help seeking adolescents. Evidence-based mental health 2012.

Griffiths KM, Christensen H. Internet-based mental health programs: A powerful tool in the rural medical kit. Aust J Rural Health 2007;15:81-87.

Griffiths KM, Farrer L, Christensen H. The efficacy of internet interventions for depression and anxiety disorders: A review of randomised controlled trials (structured abstract). Medical Journal of Australia [serial on the Internet]. 2010; (11 Supplement): Available from: http://www.mrw.interscience.wiley.com/cochrane/cldare/articles/DARE-12010006160/frame.html.

Hayes, Inc. Computer-assisted cognitive-behavioral treatment for depression (structured abstract). Lansdale, PA: HAYES, Inc [serial on the Internet]. 2009: Available from: http://www.mrw.interscience.wiley.com/cochrane/clhta/articles/HTA-32010000852/frame.html.

Hedman E, Ljotsson B, Lindefors N. Cognitive behavior therapy via the internet: A systematic review of applications, clinical efficacy and cost-effectiveness. Expert Rev Pharmacoecon Outcomes Res 2012;12:745-764.

Herbst N, Voderholzer U, Stelzer N, et al. The potential of telemental health applications for obsessive–compulsive disorder. Clin Psychol Rev 2012;32:454-466.

Ingram D, Moreno M. A computerized self-help intervention is as effective as face-to-face counseling for adolescents seeking help for depression. J Pediatr 2012;161:967-968.

Johansson R, Andersson G. Internet-based psychological treatments for depression. Expert Rev Neurother 2012;12:861-870.

Kaltenthaler E, Parry G, Beverley C, Ferriter M. Computerised cognitive-behavioural therapy for depression: Systematic review. Br J Psychiatry 2008;193:181-184.

Kaltenthaler E, Sutcliffe P, Parry G, et al. The acceptability to patients of computerized cognitive behaviour therapy for depression: A systematic review. Psychol Med 2008;38:1521-1530.

Lanche M, Perkins C, Jr., Stoltzfoos L. Live, online cbt helps service members with ptsd. Prim Psychiatry 2008;15:20.

Mundy L, Hiller JE. Internet delivered cognitive behavioural therapy for patients with depression. Adelaide: Adelaide Health Technology Assessment (AHTA). National Horizon Scanning Summary Volume 25. 2009.

Newall C, Hudson JL. Online cognitive-behaviour therapy is similarly effective to clinic-based cbt for reducing adolescent anxiety. Evid Based Ment Health 2012;15:49.

Reger MA, Gahm GA. A meta-analysis of the effects of internet- and computer-based cognitive-behavioral treatments for anxiety. J Clin Psychol. 2009;65:53-75.

Richards D, Richardson T. Computer-based psychological treatments for depression: A systematic review and meta-analysis. Clin Psychol Rev 2012;32:329-342.

Richardson T, Stallard P, Velleman S. Computerised cognitive behavioural therapy for the prevention and treatment of depression and anxiety in children and adolescents: A systematic review. Clin Child Fam Psychol Rev 2010;13:275-290.

Spek V, Cuijpers P, Nyklicek I, et al. Internet-based cognitive behaviour therapy for symptoms of depression and anxiety: A meta-analysis. Psychol Med 2007;37:319-328.

Stuhlmiller C, Tolchard B. Computer-assisted cbt for depression & anxiety: Increasing accessibility to evidence-based mental health treatment. J Psychosoc Nurs Ment Health Serv 2009;47:32-39.

Tumur I, Kaltenthaler E, Ferriter M, et al. Computerised cognitive behaviour therapy for obsessive-compulsive disorder: A systematic review. Psychother Psychosom 2007;76:196-202.

Wade AG. Use of the internet to assist in the treatment of depression and anxiety: A systematic review. Prim Care Companion J Clin Psychiatry 2010;12.

Waller R, Gilbody S. Barriers to the uptake of computerized cognitive behavioural therapy: A systematic review of the quantitative and qualitative evidence. Psychol Med 2009;39:705-712.

## Research question not relevant, 40 reports

Anderson RE, Spence SH, Donovan CL, et al. Working alliance in online cognitive behavior therapy for anxiety disorders in youth: Comparison with clinic delivery and its role in predicting outcome. J Med Internet Res 2012;14:e88.

Andersson G, Bergström J, Holländare F, et al. Delivering cognitive behavioural therapy for mild to moderate depression via the internet: Predicting outcome at 6-month follow-up [behavior therapy cognitive internetbasierte fiir easy until middle depression: Prediction of the therapy success after 6-monatiger katamnese]. Verhaltenstherapie 2004;14:185-189.

Andersson G, Paxling B, Wiwe M, et al. Therapeutic alliance in guided internet-delivered cognitive behavioural treatment of depression, generalized anxiety disorder and social anxiety disorder. Behav Res Ther 2012;50:544-550.

Berger T, Caspar F, Richardson R, et al. Internet-based treatment of social phobia: A randomized controlled trial comparing unguided with two types of guided self-help. Behav Res Ther 2011;49:158-169.

Boettcher J, Berger T, Renneberg B. Does a pre-treatment diagnostic interview affect the outcome of internet-based self-help for social anxiety disorder? A randomized controlled trial. Behav Cogn Psychother 2012;40:513-528.

Button KS, Wiles NJ, Lewis G, et al. Factors associated with differential response to online cognitive behavioural therapy. Soc Psychiatry Psychiatr Epidemiol 2012;47:827-833.

Carlbring P, Ekselius L, Andersson G. Treatment of panic disorder via the internet: A randomized trial of cbt vs. applied relaxation. J Behav Ther Exp Psychiatry 2003;34:129-140.

Carter FA, Bell CJ, Colhoun HC. Suitability and acceptability of computerised cognitive behaviour therapy for anxiety disorders in secondary care. Aust N Z J Psychiatry 2013;47:142-152.

Christensen H, Griffiths KM, Mackinnon AJ, Brittliffe K. Online randomized controlled trial of brief and full cognitive behaviour therapy for depression. Psychol Med 2006;36:1737-1746.

de Graaf LE, Hollon SD, Huibers MJ. Predicting outcome in computerized cognitive behavioral therapy for depression in primary care: A randomized trial. J Consult Clin Psychol 2010;78:184-189.

de Graaf LE, Huibers MJ, Riper H, et al. Use and acceptability of unsupported online computerized cognitive behavioral therapy for depression and associations with clinical outcome. J Affect Disord 2009;116:227-231.

Donkin L, Hickie IB, Christensen H, et al. Sampling bias in an internet treatment trial for depression. Transl Psychiatry 2012;2:e174.

Eisdorfer C, Czaja SJ, Loewenstein DA, et al. The effect of a family therapy and technology-based intervention on caregiver depression. The Gerontologist 2003;43:521-531.

Gega L, Smith J, Reynolds S. Cognitive behaviour therapy (cbt) for depression by computer vs. Therapist: Patient experiences and therapeutic processes. Psychother Res 2013;23:218-231.

Health Technology Assessment. The randomised evaluation of the effectiveness and acceptability of computerised therapy (reeact) trial (project record). Health Technology Assessment 2010: Available from: http://www.nets.nihr.ac.uk/projects/hta/064305.

Hilvert-Bruce Z, Rossouw PJ, Wong N, et al. Adherence as a determinant of effectiveness of internet cognitive behavioural therapy for anxiety and depressive disorders. Behav Res Ther 2012;50:463-468.

Hoek W, Aarts F, Schuurmans J, Cuijpers P. Who are we missing? Non-participation in an internet intervention trial for depression and anxiety in adolescents. Eur Child Adolesc Psychiatry 2012.

Hoek W, Marko M, Fogel J, et al. Randomized controlled trial of primary care physician motivational interviewing versus brief advice to engage adolescents with an internet-based depression prevention intervention: 6-month outcomes and predictors of improvement. Transl Res 2011;158:315-325.

Kenter R, Warmerdam L, Brouwer-Dudokdewit C, et al. Guided online treatment in routine mental health care: An observational study on uptake, drop-out and effects. BMC Psychiatry 2013;13:43.

Klein B, Austin D, Pier C, et al. Internet-based treatment for panic disorder: Does frequency of therapist contact make a difference? Cogn Behav Ther 2009;38:100-113.

Knaevelsrud C, Liedl A, Maercker A. Posttraumatic growth, optimism and openness as outcomes of a cognitive-behavioural intervention for posttraumatic stress reactions. J Health Psychol 2010;15:1030-1038.

Lauder S, Chester A, Castle D, et al. Development of an online intervention for bipolar disorder. Www.Moodswings.Net.Au. Psychol Health Med 2013;18:155-165.

Lorian CN, Titov N, Grisham JR. Changes in risk-taking over the course of an internet-delivered cognitive behavioral therapy treatment for generalized anxiety disorder. J Anxiety Disord 2012;26:140-149.

Morgan AJ, Mackinnon AJ, Jorm AF. Behavior change through automated e-mails: Mediation analysis of self-help strategy use for depressive symptoms. Behav Res Ther 2013;51:57-62.

Moritz S, Schroder J, Meyer B, Hauschildt M. The more it is needed, the less it is wanted: Attitudes toward face-to-face intervention among depressed patients undergoing online treatment. Depress Anxiety. 2012;30:157-167.

Poole R, Simpson SA, Smith DJ. Internet-based psychoeducation for bipolar disorder: A qualitative analysis of feasibility, acceptability and impact. BMC Psychiatry 2012;12:139.

Proudfoot J, Parker G, Manicavasagar V, et al. Effects of adjunctive peer support on perceptions of illness control and understanding in an online psychoeducation program for bipolar disorder: A randomised controlled trial. J Affect Disord 2012.

Quilty LC, McBride C, Bagby RM. Evidence for the cognitive mediational model of cognitive behavioural therapy for depression. Psychol Med 2008;38:1531-1541.

Richards DL. Client-identified helpful and hindering events in therapist-delivered vs. Self-administered online cognitive-behavioural treatments for depression in college students. Couns Psychol Q 2012;25:251-262.

Schneider AJ, Mataix-Cols D, Marks IM, Bachofen M. Internet-guided self-help with or without exposure therapy for phobic and panic disorders. Psychother Psychosom 2005;74:154-164.

Simon GE, Ludman EJ, Goodale LC, et al. An online recovery plan program: Can peer coaching increase participation? Psychiatr Serv 2011;62:666-669.

Tillfors M, Carlbring P, Furmark T, et al. Treating university students with social phobia and public speaking fears: Internet delivered self-help with or without live group exposure sessions. Depress Anxiety 2008;25:708-717.

Titov N, Andrews G, Schwencke G, et al. Randomized controlled trial of internet cognitive behavioural treatment for social phobia with and without motivational enhancement strategies. Aust N Z J Psychiatry 2010;44:938-945.

Titov N, Andrews G, Schwencke G, et al. An rct comparing effect of two types of support on severity of symptoms for people completing internet-based cognitive behaviour therapy for social phobia. Aust N Z J Psychiatry 2009;43:920-926.

Van Voorhees BW, Fogel J, Pomper BE, et al. Adolescent dose and ratings of an internet-based depression prevention program: A randomized trial of primary care physician brief advice versus a motivational interview. J Cogn Behav Psychother 2009;9:1-19.

Van Voorhees BW, Fogel J, Reinecke MA, et al. Randomized clinical trial of an internet-based depression prevention program for adolescents (project catch-it) in primary care: 12-week outcomes. J Dev Behav Pediatr 2009;30:23-37.

Van Voorhees BW, Vanderplough-Booth K, Fogel J, et al. Integrative internet-based depression prevention for adolescents: A randomized clinical trial in primary care for vulnerability and protective factors. J Can Acad Child Adolesc Psychiatry 2008;17:184-196.

Warmerdam L, van Straten A, Jongsma J, et al. Online cognitive behavioral therapy and problem-solving therapy for depressive symptoms: Exploring mechanisms of change. J Behav Ther Exp Psychiatry 2010;41:64-70.

Watts S, Mackenzie A, Thomas C, et al. Cbt for depression: A pilot rct comparing mobile phone vs. Computer. BMC Psychiatry 2013;13:49.

Yellowlees PM, Holloway KM, Parish MB. Therapy in virtual environments--clinical and ethical issues. Telemed J E Health 2012;18:558-564.

## Intervention not via Internet or otherwise not eligible, 35 reports

Amir N, Taylor CT. Interpretation training in individuals with generalized social anxiety disorder: A randomized controlled trial. J Consult Clin Psychol 2012.

Beard C, Weisberg RB, Amir N. Combined cognitive bias modification treatment for social anxiety disorder: A pilot trial. Depress Anxiety 2011;28:981-988.

Bowler JO, Mackintosh B, Dunn BD, et al. A comparison of cognitive bias modification for interpretation and computerized cognitive behavior therapy: Effects on anxiety, depression, attentional control, and interpretive bias. J Consult Clin Psychol 2012.

Cukrowicz KC, Joiner Jr TE. Computer-based intervention for anxious and depressive symptoms in a non-clinical population. Cognit Ther Res. 2007;31:677-693.

Dewis LM, Kirkby KC, Martin F, et al. Computer-aided vicarious exposure versus live graded exposure for spider phobia in children. J Behav Ther Exp Psychiatry 2001;32:17-27.

Fledderus M, Bohlmeijer ET, Pieterse ME, Schreurs KM. Acceptance and commitment therapy as guided self-help for psychological distress and positive mental health: A randomized controlled trial. Psychol Med 2012;42:485-495.

Fleming T, Dixon R, Frampton C, Merry S. A pragmatic randomized controlled trial of computerized cbt (sparx) for symptoms of depression among adolescents excluded from mainstream education. Behav Cogn Psychother 2011:1-13.

Gorini A, Pallavicini F, Algeri D, et al. Virtual reality in the treatment of generalized anxiety disorders. Studies in health technology and informatics 2010;154:39-43.

Hazen RA, Vasey MW, Schmidt NB. Attentional retraining: A randomized clinical trial for pathological worry. J Psychiatr Res 2009;43:627-633.

Kay-Lambkin F, Baker A, Lewin T, Carr V. Acceptability of a clinician-assisted computerized psychological intervention for comorbid mental health and substance use problems: Treatment adherence data from a randomized controlled trial. J Med Internet Res 2011;13:e11.

Kay-Lambkin FJ, Baker AL, Kelly B, Lewin TJ. Clinician-assisted computerised versus therapist-delivered treatment for depressive and addictive disorders: A randomised controlled trial. Med J Aust 2011;195:S44-50.

Kay-Lambkin FJ, Baker AL, Lewin TJ, Carr VJ. Computer-based psychological treatment for comorbid depression and problematic alcohol and/or cannabis use: A randomized controlled trial of clinical efficacy. Addiction 2009;104:378-388.

Kenardy JA, Dow MG, Johnston DW, et al. A comparison of delivery methods of cognitive-behavioral therapy for panic disorder: An international multicenter trial. J Consult Clin Psychol 2003;71:1068-1075.

Kenwright M, Marks I, Graham C, et al. Brief scheduled phone support from a clinician to enhance computer-aided sell-help for obsessive-compulsive disorder: Randomized controlled trial. J Clin Psychol 2005;61:1499-1508.

Khanna MS, Kendall PC. Computer-assisted cognitive behavioral therapy for child anxiety: Results of a randomized clinical trial. J Consult Clin Psychol 2010;78:737-745.

Levesque DA, Van Marter DF, Schneider RJ, et al. Randomized trial of a computer-tailored intervention for patients with depression. Am J Health Promot 2011;26:77-89.

Levin W, Campbell DR, McGovern KB, et al. A computer-assisted depression intervention in primary care. Psychol Med 2011;41:1373-1383.

Marchand A, Beaulieu-Prévost D, Guay S, et al. Relative efficacy of cognitive-behavioral therapy administered by videoconference for posttraumatic stress disorder: A six-month follow-up. J Aggress Maltreat Trauma 2011;20:304-321.

Marks IM, Kenwright M, McDonough M, et al. Saving clinicians' time by delegating routine aspects of therapy to a computer: A randomized controlled trial in phobia/panic disorder. Psychol Med 2004;34:9-17.

Merry SN, Stasiak K, Shepherd M, et al. The effectiveness of sparx, a computerised self help intervention for adolescents seeking help for depression: Randomised controlled non-inferiority trial. BMJ 2012;344:1-16.

Moreno FA, Chong J, Dumbauld J, et al. Use of standard webcam and internet equipment for telepsychiatry treatment of depression among underserved hispanics. Psychiatr Serv 2012;63:1213-1217.

Muller BH, Kull S, Wilhelm FH, Michael T. One-session computer-based exposure treatment for spider-fearful individuals--efficacy of a minimal self-help intervention in a randomised controlled trial. J Behav Ther Exp Psychiatry 2011;42:179-184.

Newman MG, Kenardy J, Herman S, Taylor CB. Comparison of palmtop-computer-assisted brief cognitive-behavioral treatment to cognitive-behavioral treatment for panic disorder. J Consult Clin Psychol 1997;65:178-183.

Possemato K, Ouimette P, Knowlton P. A brief self-guided telehealth intervention for post-traumatic stress disorder in combat veterans: A pilot study. J Telemed Telecare 2011;17:245-250.

Preschl B, Maercker A, Wagner B, et al. Life-review therapy with computer supplements for depression in the elderly: A randomized controlled trial. Aging Ment Health 2012.

Proudfoot J, Goldberg D, Mann A, et al. Computerized, interactive, multimedia cognitive-behavioural program for anxiety and depression in general practice. Psychol Med 2003;33:217-227.

Seligman ME, Schulman P, Tryon AM. Group prevention of depression and anxiety symptoms. Behav Res Ther 2007;45:1111-1126.

Sethi S, Campbell AJ, Ellis LA. The use of computerized self-help packages to treat adolescent depression and anxiety. J Technol Hum Serv 2010;28:144-160.

Spence SH, Holmes JM, March S, Lipp OV. The feasibility and outcome of clinic plus internet delivery of cognitive-behavior therapy for childhood anxiety. J Consult Clin Psychol 2006;74:614-621.

Stallard P, Richardson T, Velleman S, Attwood M. Computerized cbt (think, feel, do) for depression and anxiety in children and adolescents: Outcomes and feedback from a pilot randomized controlled trial. Behav Cogn Psychother 2011;39:273-284.

Stasiak K, Hatcher S, Frampton C, Merry SN. A pilot double blind randomized placebo controlled trial of a prototype computer-based cognitive behavioural therapy program for adolescents with symptoms of depression. Behav Cogn Psychother 2012:1-17.

Tortella-Feliu M, Botella C, Llabres J, et al. Virtual reality versus computer-aided exposure treatments for fear of flying. Behav Modif 2011;35:3-30.

Wright JH, Wright AS, Albano AM, et al. Computer-assisted cognitive therapy for depression: Maintaining efficacy while reducing therapist time. Am J Psychiatry 2005;162:1158-1164.

Wuthrich VM, Rapee RM, Cunningham MJ, et al. A randomized controlled trial of the cool teens cd-rom computerized program for adolescent anxiety. J Am Acad Child Adolesc Psychiatry 2012;51:261-270.

Zautra AJ, Davis MC, Reich JW, et al. Phone-based interventions with automated mindfulness and mastery messages improve the daily functioning for depressed middle-aged community residents. J Psychother Integr 2012;22:206-228.

## Research design, 7 reports

Hunkeler EM, Hargreaves WA, Fireman B, et al. A web-delivered care management and patient self-management program for recurrent depression: A randomized trial. Psychiatr Serv 2012;63:1063-1071.

Mewton L, Wong N, Andrews G. The effectiveness of internet cognitive behavioral therapy for generalized anxiety disorder in clinical practice. Depress Anxiety 2012;29:843-849.

Pier C, Austin DW, Klein B, et al. A controlled trial of internet-based cognitive-behavioural therapy for panic disorder with face-to-face support from a general practitioner or email support from a psychologist. Ment Health Fam Med 2008;5:29-39.

Pittaway S, Cupitt C, Palmer D, et al. Comparative, clinical feasibility study of three tools for delivery of cognitive behavioural therapy for mild to moderate depression and anxiety provided on a self-help basis. Mental Health in Family Medicine 2009;6:145-154.

Proudfoot J, Ryden C, Everitt B, et al. Clinical efficacy of computerised cognitive-behavioural therapy for anxiety and depression in primary care: Randomised controlled trial. Br J Psychiatry 2004;185:46-54.

Ruwaard J, Lange A, Schrieken B, et al. The effectiveness of online cognitive behavioral treatment in routine clinical practice. PLoS One 2012;7:e40089.

Williams AD, Andrews G. The effectiveness of internet cognitive behavioural therapy (icbt) for depression in primary care: A quality assurance study. PLoS One 2013;8:e57447.

## Protocol, 2 reports

Barnes C, Harvey R, Mitchell P, et al. Evaluation of an online relapse prevention program for bipolar disorder: An overview of the aims and methodology of a randomized controlled trial. Disease Management & Health Outcomes 2007;15:215-224.

van der Zanden RA, Kramer JJ, Cuijpers P. Effectiveness of an online group course for adolescents and young adults with depressive symptoms: Study protocol for a randomized controlled trial. Trials 2011;12:196.

# Efficacy trials with high risk of bias, 13 reports

## Mood disorders

Andersson G, Hesser H, Hummerdal D, et al. A 3.5-year follow-up of internet-delivered cognitive behavior therapy for major depression. J Ment Health 2011.

Perini S, Titov N, Andrews G. Clinician-assisted internet-based treatment is effective for depression: Randomized controlled trial. Aust N Z J Psychiatry 2009;43:571-578.

## Social phobia

Andrews G, Davies M, Titov N. Effectiveness randomized controlled trial of face to face versus internet cognitive behaviour therapy for social phobia. Aust N Z J Psychiatry 2011;45:337-340.

Botella C, Gallego MJ, Garcia-Palacios A, et al. An internet-based self-help treatment for fear of public speaking: A controlled trial. Cyberpsychol Behav Soc Netw 2010;13:407-421.

Titov N, Andrews G, Choi I, et al. Randomized controlled trial of web-based treatment of social phobia without clinician guidance. Aust N Z J Psychiatry 2009;43:913-919.

## Panic disorder

Carlbring P, Westling BE, Ljungstrand P, et al. Treatment of panic disorder via the internet: A randomized trial of a self-help program. Behav Ther 2001;32:751-764.

Kiropoulos LA, Klein B, Austin DW, et al. Is internet-based cbt for panic disorder and agoraphobia as effective as face-to-face cbt? J Anxiety Disord 2008;22:1273-1284.

Klein B, Richards JC. A brief internet-based treatment for panic disorder. Behav Cogn Psychother 2001;29:113-117.

Richards JC, Klein B, Austin DW. Internet cognitive behavioural therapy for panic disorder: Does the inclusion of stress management information improve end-state functioning? Clinical Psychologist 2006;10:2-15.

## Posttraumatic stress disorder

Litz BT, Engel CC, Bryant RA, Papa A. A randomized, controlled proof-of-concept trial of an internet-based, therapist-assisted self-management treatment for posttraumatic stress disorder. Am J Psychiatry 2007;164:1676-1683.

## Children and adolescents

Spence SH, Donovan CL, March S, et al. A randomized controlled trial of online versus clinic-based cbt for adolescent anxiety. J Consult Clin Psychol 2011;79:629-642.

Storch EA, Caporino NE, Morgan JR, et al. Preliminary investigation of web-camera delivered cognitive-behavioral therapy for youth with obsessive-compulsive disorder. Psychiatry research 2011;189:407-412.

Tillfors M, Andersson G, Ekselius L, et al. A randomized trial of internet-delivered treatment for social anxiety disorder in high school students. Cogn Behav Ther 2011;40:147-157.

# Irrelevant cost-effectiveness studies, 13 reports

de Graaf LE, Gerhards SA, Arntz A, et al. One-year follow-up results of unsupported online computerized cognitive behavioural therapy for depression in primary care: A randomized trial. J Behav Ther Exp Psychiatry 2011;42:89-95.

Gerhards SA, de Graaf LE, Jacobs LE, et al. Economic evaluation of online computerised cognitive-behavioural therapy without support for depression in primary care: Randomised trial. Br J Psychiatry 2010;196:310-318.

Griffiths KM, Christensen H. Internet-based mental health programs: A powerful tool in the rural medical kit. Aust J Rural Health 2007;15:81-87.

Heilman RM, Kállay É, Miclea M. The role of computer-based psychotherapy in the treatment of anxiety disorders. Cogn Brain Behav 2010;14:209-230.

Hoek W, Schuurmans J, Koot HM, Cuijpers P. Prevention of depression and anxiety in adolescents: A randomized controlled trial testing the efficacy and mechanisms of internet-based self-help problem-solving therapy. Trials 2009;10:93.

Kaltenthaler E, Brazier J, De Nigris E, et al. Computerised cognitive behaviour therapy for depression and anxiety update: A systematic review and economic evaluation. Health Technol Assess 2006;10:iii, xi-xiv, 1-168.

Lewis C, Pearce J, Bisson JI. Efficacy, cost-effectiveness and acceptability of self-help interventions for anxiety disorders: Systematic review. Br J Psychiatry 2012;200:15-21.

McCrone P, Knapp M, Proudfoot J, et al. Cost-effectiveness of computerised cognitive-behavioural therapy for anxiety and depression in primary care: Randomised controlled trial. Br J Psychiatry 2004;185:55-62.

McCrone P, Marks IM, Greist JH, et al. Cost-effectiveness of computer-aided behaviour therapy for obsessive-compulsive disorder. Psychother Psychosom 2007;76:249-250.

McCrone P, Marks IM, Mataix-Cols D, et al. Computer-aided self-exposure therapy for phobia/panic disorder: A pilot economic evaluation. Cogn Behav Ther 2009;38:91-99.

Newman MG, Szkodny LE, Llera SJ, Przeworski A. A review of technology-assisted self-help and minimal contact therapies for anxiety and depression: Is human contact necessary for therapeutic efficacy? Clin Psychol Rev 2011;31:89-103.

Palmqvist B, Carlbring P, Andersson G. Internet-delivered treatments with or without therapist input: Does the therapist factor have implications for efficacy and cost? Expert Rev Pharmacoecon Outcomes Res 2007;7:291-297.

Warmerdam L, Smit F, van Straten A, et al. Cost-utility and cost-effectiveness of internet-based treatment for adults with depressive symptoms: Randomized trial. J Med Internet Res 2010;12:e53.

# Cost-effectiveness studies with high risk of bias, 3 reports

Bergstrom J, Andersson G, Ljotsson B, et al. Internet-versus group-administered cognitive behaviour therapy for panic disorder in a psychiatric setting: A randomised trial. BMC Psychiatry 2010;10:54.

Mihalopoulos C, Kiropoulos L, Shih ST, et al. Exploratory economic analyses of two primary care mental health projects: Implications for sustainability. Med J Aust 2005;183:S73-76.

Titov N, Andrews G, Johnston L, et al. Shyness programme: Longer term benefits, cost-effectiveness, and acceptability. Aust N Z J Psychiatry 2009;43:36-44.
