# Supplementary material for: Internet-Delivered Psychological Treatments for Mood and Anxiety Disorders: A Systematic Review of Their Efficacy, Safety, and Cost-Effectiveness
Source: PLoS One. 2014 May 20;9(5):e98118. doi: 10.1371/journal.pone.0098118 (PMC4028301; doi:10.1371/journal.pone.0098118)
Supplement: Protocol S1 — Study protocol. (DOCX) [file pone.0098118.s004.docx]

Internet-delivered psychological treatment for anxiety and mood disorders

# Project description

SBU ref UTV2012/26

## Background

The Swedish Council on Health Technology Assessment (SBU) released a report in 2007 regarding computer-based cognitive behavioral therapy (CBT) for anxiety disorders or depression [1]. The conclusions were that “There is limited scientific evidence that computer-based CBT, in the short term, has a favorable effect on symptoms in the treatment of panic disorder, social phobia and depression (Evidence-grading 3). The scientific basis is inadequate for evaluating efficacy of this treatment for obsessive-compulsive disorder and mixed anxiety/depression. The scientific basis is insufficient for assessing the cost-effectiveness.”

Since much new research has emerged since 2007 the present report aims to update the current knowledge. In this update the aims will be broadened to include Internet-based psychological treatments in general, and include other mood disorders apart from major depression. Unlike the previous report, this report will also include treatments for children and young people.

## Anxiety and mood disorders

Anxiety is an intensely unpleasant condition and has its origins in the uneasiness or bodily tensions that can be experienced in anticipation of an emergency or accident. A defining feature of anxiety is the lack of a real threat and that the individual’s reaction, therefore, is not appropriate. Worry and anxiety as individual symptoms are common and occur in several psychiatric disorders, such as depression and psychosis. Anxiety disorders refers to several symptoms of anxiety occurring simultaneously in a specific manner and with a specific duration. The disorders are categorized into panic disorder, agoraphobia, obsessive-compulsive disorder, post-traumatic stress disorder (PTSD), generalized anxiety disorder (GAD), social phobia, and specific phobias.

Mood disorders are psychiatric conditions characterized by lowered or elevated mood. This diagnostic category includes depressive disorders (characterized as only depressive episodes) and bipolar disorders (characterized as episodes of both mania/hypomania and depression). The core symptoms of depression include sadness and loss of interest in everyday activities, while the core symptoms of mania/hypomania are elevated, expansive or irritable mood. A diagnosis of anxiety or mood disorders is made according to the DSM- IV and ICD-10.

Anxiety disorders often has their onset in childhood or adolescence, while mood disorders tend to debut in adolescence or later [2]. It has been estimated that every four people at some time during life will suffer from an anxiety disorder [3], while every fifth person in their lifetime will suffer from a serious depression. For many, the depression becomes chronic or recurrent [4]. Anxiety and mood disorders are among the most common causes of ill health, lost productivity and work days worldwide and those affected also often have somatic and other psychiatric illnesses.

## Internet-based treatment of anxiety and mood disorders

In their guidelines for the care of depression and anxiety disorders, the National Board of Health and Welfare highlights the importance of an increased range of psychological treatment services [5]. Psychological treatment, particularly CBT, ranked high in almost all mild and moderate states of depression and anxiety disorders.

During the past decade, the development of the internet has brought new opportunities to deliver psychological treatment, and the number of randomized controlled trials (RCTs) of CBT mediated via the internet has risen sharply in recent years [6]. Internet-based treatment has the potential to a quick outreach and cater to patients who would otherwise not have received treatment. At the same time, the implementation of this form of treatment raises issues about the characteristics of the patient groups that take part in this treatment and what options that are available. Implementation in clinical practice–and the ethical questions arising from this–will therefore have a central role in the evaluation of Internet-based psychological treatment.

## Research questions

The current report will address five key themes relating to the role of Internet-based psychological treatment of anxiety and mood disorders in healthcare:
1. *Efficacy and side effects*: What effect has the internet-based psychological treatment for anxiety and mood disorders, and at sub-clinical symptoms of these disorders? What are the risks and side effects associated with treatment? How important is the degree of therapist contact for efficacy and side effects?

2. *Transferability to clinical practice*: Which patients comprise an appropriate audience for this treatment? What is the interest in this treatment in patients in routine clinical practice? What is the level of satisfaction among patients post-treatment?

3. *Implementation*: To what extent, what context, and in what way have the treatments been implemented in the Swedish healthcare? What can explain regional differences in implementation?

4. *Cost*: is internet-based psychological treatment cost-effective in comparison with the relevant treatment options?

5. *Ethics*: A number of ethical values and principles, such as humanity, integrity and confidentiality, arise with the implementation of internet-based psychological treatment. Potential value conflicts will be identified and analyzed and the results of the analysis are presented.

## Literature search

The literature search will be concern the databases PubMed, Cochrane Library, CINAL, PsycINFO, and other databases relevant to the research questions. The reference lists of the included reports will be scrutinized to identify additional relevant articles.

### Inclusion

The issue of the treatment effect will primarily be restricted to randomized controlled trials ( RCTs). For other questions, all the relevant literature, including observational and qualitative studies, will be studied. Trials are included in which the diagnosis has been made according to DSM- IV or ICD -10, or symptoms have been determined according to a rating scale. Besides treatment secondary prevention is also included (defined as elevated symptom level but subsyndromal). Follow-up time: immediately after completion of treatment and follow-up ≥ 6 months.

P- People with anxiety disorders and/or mood disorders, and those with subclinical symptoms of these disorders

I - Internet-based psychological treatments

C - waiting list, usual treatment, treatment with therapist, other treatment of anxiety disorders and/or depression, or no treatment

O - Change in symptoms, quality of life, health needs and function, effect on sick-leave, side effects, interest in participation, attrition, and costs

### Exclusion

Primary prevention and trials of defined subgroups such as patients with specific illnesses will be excluded. Also treatments that are not internet-based (e.g., CD-ROM), or based on other technologies (e.g., “Virtual Reality”) and computer-based information about the condition or treatment are excluded.

### Quality assessment

The studies that meet the inclusion criteria are assessed with the support of examination protocols and studies are graded based on their risk of bias.

### Synthesis and grading of evidence

The strength of the body of scientific evidence is assessed with the internationally developed GRADE system [7, 8], but with some adaptations. Evidence strength is categorized as strong ( + + + ), moderate ( + + + O) , limited ( + + OO) and inadequate (+ OOO ) scientific evidence.

## Mapping of services

Collection of primary data will be made to assess the extent of internet-based psychological treatment in Sweden today, describe the context in which the services are implemented and highlight experiences from everyday practice. The mapping of services will complement the literature search in answering questions 2-5.

## Structure of the report

The report will be published as an SBU Alert report, and is estimated to include approximately 15 pages. The provisional title is “Internet-based psychological treatment for anxiety and mood disorders”.

## Recipients of the results

The primary target group is policy makers in healthcare as well as politicians, administrators, clinical practitioners of different professions, relevant patient organizations, and the public.

## Marketing

The ambition is that right from the start, and continuing in various ways, spread awareness and support for the project . This will be done partly by:

• A brief description of the project posted on the SBU website.

• The report posted on the SBU website.

• Article published in the SBU periodical.

• A collaboration with *Dagens Medicin* and the *Läkartidningen* means that they probably will publish an article on the topic or comment on the report.

• The project team including the Alert council informs about the project through lectures, etc.

• Information on the project is provided under the established partnership with the County Council’s recipient organizations and relevant professional bodies.

• E- mails sent to the SBU’s subscribers made in connection with the publication of the report.

• mailings to SBU “paper subscribers.”

## Timetable

The work will be conducted based on the goal that the results should be ready for publication in the winter of 2012.

Project raised in the Alert council for approval: March 29, 2012

Results in tabular form will be completed: May 2012

First script from an expert: September 1, 2012

Draft for the economic section: September 1, 2012

Presentation of results in Alertrådet: June 12, 2012

Screenplay is sent to external examiners: autumn 2012

Alertrådet 's approval: autumn 2012

Manuscript sent for publication: autumn 2012

Publishing: winter 2012

## Publication Policy

The report is produced by SBU in collaboration with experts in the relevant fields. The results may not be distributed or published prior to publication of the report.

## Project organisation

The project will be conducted by a team consisting of external experts and collaborators at SBU’s Office.

Experts: Steven J. Linton and Ulf Jonsson

Reviewers: (to be determined)

Health economist: Emelie Heintz

Ethics: Jan Wahlström

Project Sponsors in the Alert council: Eva Lindström

Christel Bahtsevani

Project leader: Monica Hultcrantz

Assistant project leader: Ulf Jonsson

Dissemination: Ragnar Levi and Agneta Pettersson

Literature search: Hanna Olofsson

Project Assistant: Anna Granath

Other staff: Lena Wallgren (language editing and
translation)

Elin Rye Danjelsen (typesetting and publishing)

## Bindings and conflicts of interest

All experts report any eventual bindings and conflicts of interest that may affect their objectivity. This occurs before a project begins and at any changes. In questionable cases the bindings and conflicts of interest are assessed with the SBU’s director. If SBU believes that a potential expert is disqualified, he or she cannot engage as an expert or reviewer in the project.

## Approval

This project was approved by the Alert council on March 29, 2012.

## References

1. SBU (2007) Datorbaserad kognitiv beteendeterapi vid ångestsyndrom eller depression. Stockholm: Statens beredning för medicins utvärdering. SBU Alert-rapport nr 2007-03. ISSN 1652-7151. http://www.sbu.se/200703

2. Merikangas KR, Nakamura EF, Kessler RC (2009) Epidemiology of mental disorders in children and adolescents. Dialogues Clin Neurosci 11:7-20

3. Behandling av ångestsyndrom. En systematisk litteraturöversikt. SBU-rapport nr 171, 2005.

4. Behandling av depressionssjukdomar. En systematisk litteraturöversikt. SBU-rapport nr 166, 2004.

5. Socialstyrelsen (2010) Nationella riktlinjer för vård vid depression och ångestsyndrom 2010. http://www.socialstyrelsen.se/publikationer2010/2010-3-4

6. Andersson G (2010) The promise and pitfalls of the internet for cognitive behavioural therapy. BMC medicine 8:82

7. Atkins D, Best D, Briss PA, Eccles M, Falck-Ytter Y, Flottorp S, et al. Grading quality of evidence and strength of recommendations. BMJ 2004;328:1490.

8. Guyatt G, Oxman AD, Akl EA, Kunz R, Vist G, Brozek J, et al. GRADE guidelines 1. Introduction – GRADE evidence profiles and summary of findings tables. J Clin Epidemiol 2011;64(4):383-94.
